# DNA Hydrogel-Interfaced Organic Electrochemical Transistor for the Investigation of Binding-Induced Conformational Change of Small Molecule Aptamers

**DOI:** 10.1021/acsami.5c11113

**Published:** 2025-09-08

**Authors:** Haosi Lin, Zibin Zhao, Xianzhen Feng, Sin Yu Yeung, I-Ming Hsing

**Affiliations:** Department of Chemical and Biological Engineering, The Hong Kong University of Science and Technology, Kowloon, Hong Kong 999077, China

**Keywords:** OECT, small molecule-aptamer, aptamer binding mode, DNA hydrogel, label-free biosensing

## Abstract

Investigation of the small molecule-aptamer interaction is difficult, and it usually lacks information about the conformational change of aptamers that is important for their application. Here, we present the label-free investigation of small molecule-aptamer interactions using a modularized organic electrochemical transistor (OECT) platform. Leveraging the high sensitivity of the OECT, we measured the conformational change of the aptamer encountering its ligand. This platform consists of a simply fabricated OECT device and a DNA hydrogel-based bioreceptor module. Using this device, we investigated four DNA aptamers binding to cortisol (362.46 Da) and testosterone (288.4 Da). We demonstrated the correlation between the aptamer conformational change and the OECT response. We further calculated the apparent affinities of these aptamers based on the responses. This OECT-based platform also showed potential in binding kinetic measurements. We anticipate that the presented platform could advance the application of the newly identified aptamers as a convenient assay, providing multiple metrics for characterizing small molecule-aptamer interactions.

## Graphical abstract

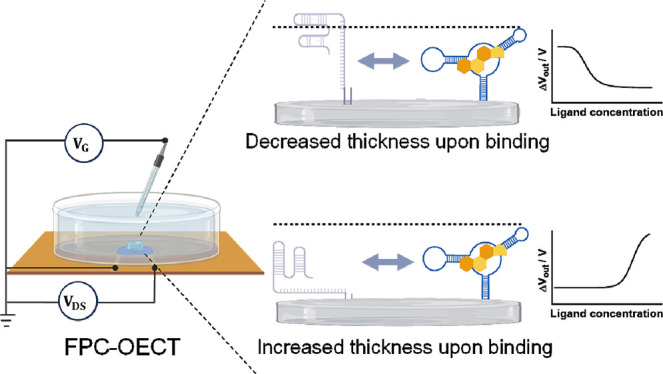

## Introduction

Aptamers are oligonucleotides that bind to a cognate target. They have shown potential in analysis, diagnostics, and therapeutics, thanks to their advantages including sensitivity, selectivity, stability, and ease of synthesis, compared to other widely used bioreceptors like antibodies and enzymes.^1^ Aptamers can be selected for a wide range of targets, including small molecules, through an iterative workflow called systematic evolution of ligands by exponential enrichment (SELEX).^2–5^ After the selection, application of aptamers requires characterization of aptamer-ligand interaction in different metrics, such as equilibrium binding affinity, binding kinetics and aptamer conformational change.^6–8^ As illustrated in Figure 1, based on the difference in the change of the conformational equilibrium of the aptamer induced by interaction with its ligand, the ligand-aptamer interaction can be classified into two modes: conformational selection and induced-fit.^9^ Conformational change of the aptamer strands induced by their interaction with the ligands in the induce-fit mode of binding could play an important role in various fields, including signal transduction design for biosensors and development of therapeutic strategies.^10–12^ Hence, identifying the binding mode of an aptamer after its discovery is necessary, as it provides important information for its applications.

**Figure 1:**
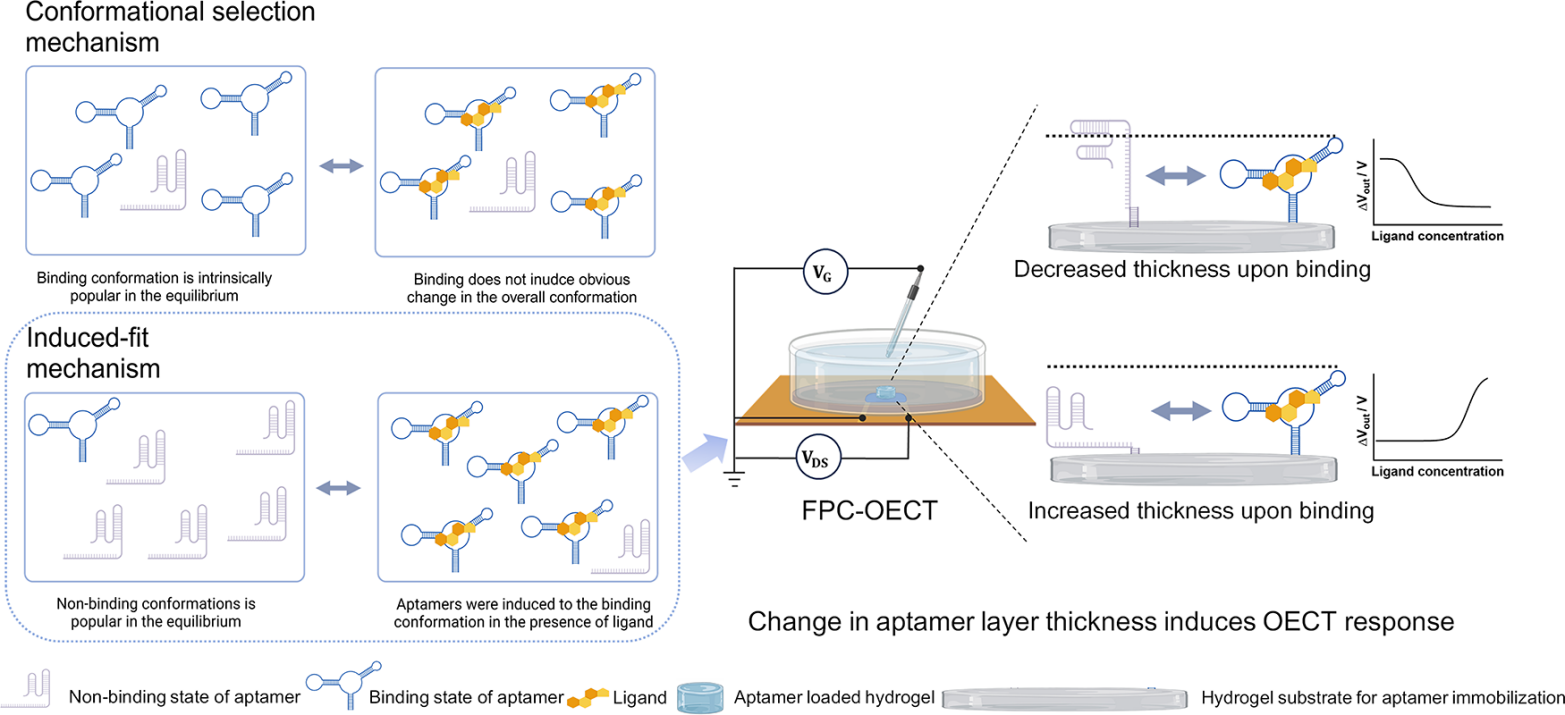
Conformational change of the aptamer in the induce-fit binding mechanism induces a response in OECT. Conformational selection mechanism: the ligand “selects” a popularly pre-existing conformation of the aptamer; induce-fit mechanism: the ligand “fits” a certain conformation of the aptamer and hence “induces” the equilibrium to shift toward the preferred conformation.

Analysis of small molecule-aptamer interaction is relatively difficult, due to the small molecular weight of the ligands.^3,6^ The commonly used analytical techniques for aptamer characterization, like surface plasmon resonance (SPR) and isothermal titration calorimetry (ITC), mainly focus on the analysis of binding affinity and kinetics, while their performance is limited in the case of small molecule aptamers.^6,13,14^ Conformation analysis tools like circular dichroism (CD) have also been used for the conformation analysis of aptamers,^8,15^ while the binding thermodynamic parameters like the binding heat capacity change (ΔC) obtained by ITC also give an insight into the conformational equilibria of aptamers upon binding.^16–18^ However, the conformational analysis using these methods requires a large amount of the sample and repeated trials, while their result interpretations are time-consuming and rely on the experience of the specialists, especially in the case of small molecule aptamers.^8^ To date, only when corresponding evidence is concurrently obtained from multiple analysis methods can the binding mode of an aptamer be revealed.^9,17^ Moreover, the specialized instruments for these techniques are usually costly and not commonly available in biological laboratories where aptamers are developed and applied.^19^ Accurate yet accessible methods for the characterization of small molecule aptamers providing conformational information are highly in demand for their development and application.

Here, we attempt to use the organic electrochemical transistor (OECT) as a platform to investigate small molecule-aptamer interaction, not only for the apparent binding affinity but also for the identification of the aptamer conformational change during binding (Figure 1). OECT is an ionic-electronic signal transducer compatible with aqueous environments, and it is sensitive to capacitance and potential changes.^20^ Therefore, it has been applied for nonfaradic biosensing, like cell impedance biosensing^21–23^ and DNA detection.^24^ We anticipate that the high sensitivity of the OECT could realize direct signal transduction for the occurrence of the induce-fit binding of an aptamer, eliminating the need for extra signal amplification reaction or redox probe in the system. Aiming at the OECT-based investigation of aptamer binding modes and apparent affinities, we first developed a simple fabrication workflow for the OECT devices based on a widely used flexible printed circuit (FPC) with an optimized channel material. Then, we designed a stand-alone ssDNA-polyacrylamide (PAAM) hydrogel-based bioreceptor module to avoid harsh treatment on the OECT device, which enabled the straightforward hybridization-based immobilization of the tested aptamers. The interconnected porous structure of the hydrogel provides more active sites for aptamer-ligand interaction, thus enabling sufficient sensitivity for the label-free signal transduction. This modularized design allows analysis of different aptamers by applying a bioreceptor module loaded with different aptamers on the same OECT device.

At the first stage, we applied a previously reported cortisol aptamer^25^ on the presented platform. A shift in the OECT transfer curve was induced by the addition of the ligand due to the conformational change of the aptamer strand, which was further verified with two other published aptamers. Ligand-aptamer interactions that introduce different changes in the aptamer layer thickness were identified through the different OECT responses (Figure 1), as verified by electrochemical impedance spectroscopy (EIS). Notably, only ligand-aptamer interactions that showed induced-fit properties could induce the OECT response in our platform. Such correlation between binding-induced conformational change and OECT response was also validated with two other reported aptamers binding with cortisol and testosterone, respectively.^26^ We further used this system to measure the apparent binding affinities (*K*_D_) of these reported sequences, along with that of another self-selected cortisol aptamer. Moreover, we demonstrated the potential of this FPC-based OECT platform in binding kinetic measurement using a handcrafted flow cell. These results support that the presented DNA hydrogel-based OECT platform can measure the conformational change induced by small molecule-aptamer interactions and potentially serve as an easily accessible characterization platform for aptamers. This could help in the development of novel aptamers for applications requiring significant conformational change upon target interactions, like the use of aptamers as regulatory elements of genetic circuits.^12^

## Results and Discussion

### Development of Stable and Easy-to-Fabricate OECT Based on FPC

First, we aimed at developing a simple fabrication workflow for the OECT devices based on the widely used FPCs. The fabrication procedure only includes spin-coating of the conducting polymer membrane on a customized FPC and a peel-off operation of the sacrificial polyimide tape to form the OECT channel. A conventional formulation of Poly­(3,4-ethylenedioxythiophene):dextran sulfate (PEDOT:PSS) membrane was first used for the FPC-based OECTs.^27^ Due to the resolution in FPC manufacturing, a relatively large channel size (width × length = 100 × 130 μm) was used for the FPC-based OECT. Such a large channel size achieves a sufficiently high transconductance (Figure S1) but compromises the response speed and stability of the device, which was also observed in our previous works involving larger channel sizes.^21^ One of the main causes of the instability of OECT in repeated operations is the swelling of the PEDOT:PSS membrane due to water intake (Figure S2A), which was also reported previously.^28,29^ To reduce the instability of the device due to water intake, we explored hydrophobic additives like waterborne polyurethanes (WPU) in the PEDOT:PSS membrane.^30–32^ In our study, the stability of the devices was evaluated by repeated operations over a time span. In each test, the characterization and transfer curves were recorded. After each test, the devices were rinsed with deionized water and dried by baking at 60 °C for 30 min. After adding WPU, we found that the FPC-based OECT can remain stable for at least 20 days upon repeated operation of the device (Figure 2A).

**Figure 2:**
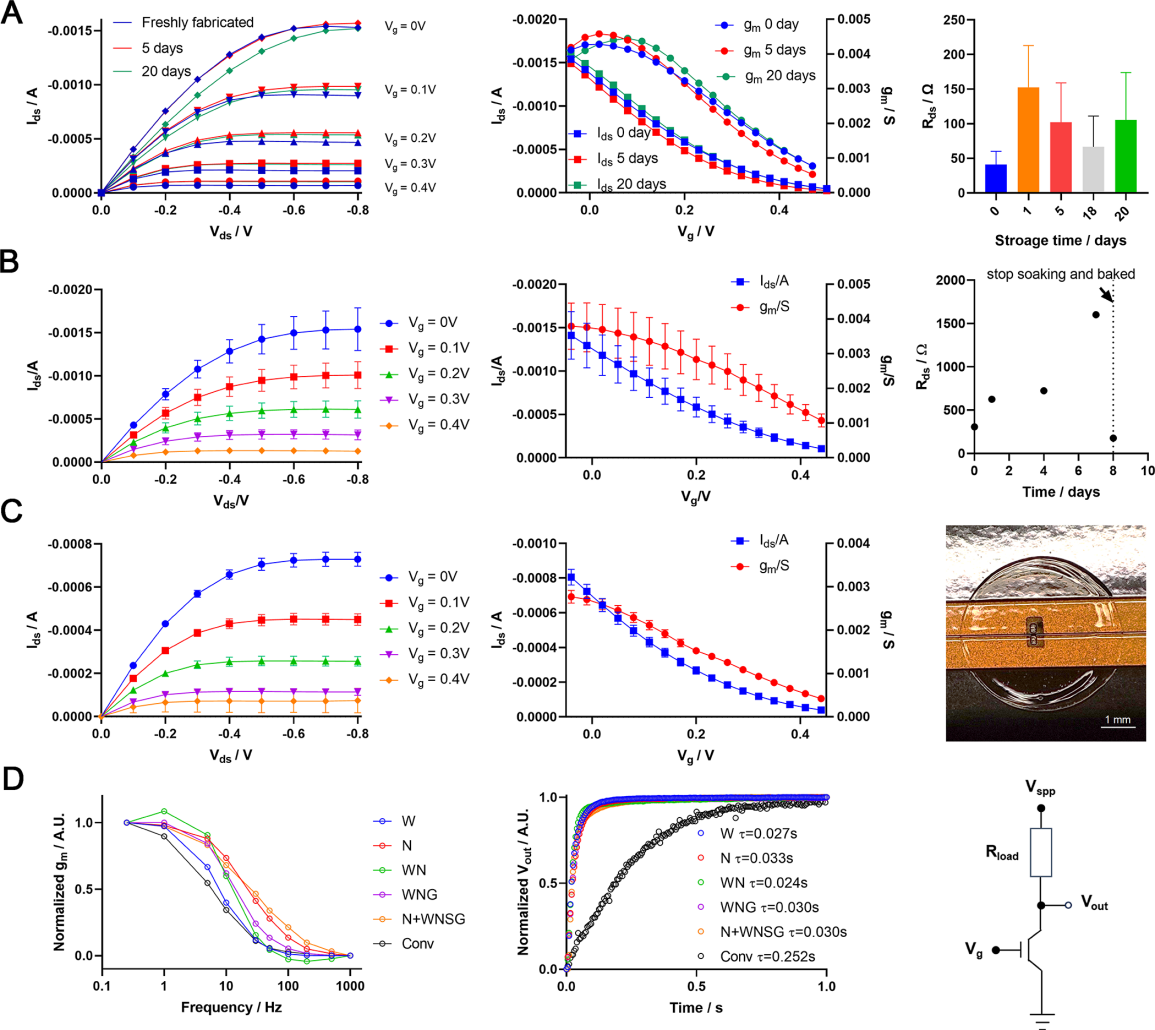
Development and optimization of the FPC-based OECT. (A) Performance and stability of WPU added FPC-based OECT evaluated by characterization curve, transfer curve and channel resistance. The error bars in the right panel are the SD of 2 devices from the same batch. (B) Performance and stability of a device using WNSG formulation. Error bars on the left and center panel show the SD of the three tests done in the 3-day trial, in which a test was done on each day. The right panel shows the channel resistance (*R*_ds_) change upon continuous soaking in PBS for 8 days. (C) Performance and stability of FPC-based OECT with a Nafion-coated channel. WNSG formulation of PEDOT:PSS membrane is used in this device. Error bars indicate the SD of the three tests done in the 12-day trial. Data on day 0, 6, and 12 were included. The right panel showed a photograph of the fabricated OECT channel. Scale bar 1 mm. (D) Frequency (left panel) and transient (center panel) response of the FPC-based OECTs with different PEDOT:PSS formulations. The right panel shows the voltage divider circuit. *R*_ds_, channel resistance; *I*_ds_, drain-source current; *V*_ds_, drain-source voltage; *V*_g_, gate voltage; *V*_spp_, supply voltage; *V*_out_, output voltage; *R*_load_, resistance load, usually set as twice the channel resistance at *V*_g_ = 0 V. W, WPU blended formulation; N, Nafion blended; WN, WPU and Nafion blended; WNG, WPU, Nafion and GOPS blended; N+WNSG, WPU, Nafion, D-sorbitol and GOPS blended, sealed with Nafion membrane, this is the optimized channel setting used in (B) and the rest of this manuscript.

In addition to the hydrophobic additives, cross-linkers and plasticizers were considered to improve the stability of the conducting polymer and further enhance the robustness of the device.^33–35^ In our study, Nafion, D-sorbitol, and (3-glycidyloxypropyl)­trimethoxysilane (GOPS) were examined. We found that the addition of Nafion, a proton-penetrable polymer containing a hydrophobic backbone, and GOPS, a typically used cross-linker for PEDOT:PSS, reduced the swelling of the membrane during the prolonged interaction with aqueous solution (Figure S2B) without compromising stability during repeated tests (Figure S3). These additives did not introduce significant changes in the morphology of the conducting polymer membranes, as visualized in scanning electrical microscopy (SEM) (Figure S4A). Among them, the addition of GOPS improved the annealing of the membrane to the polyimide substrate of the FPC (Figure S4A). D-sorbitol, a multiple hydroxyl group plasticizer that is used in our previous work,^27^ also enabled good stability upon repeated operations (Figure 2B). Notably, only the formulation containing **W**PU, **N**afion, D-**s**orbitol, and **G**OPS (the WNSG formulation) was able to retain the OECT response of the FPC-based OECT device after continuous contact with the phosphate-buffered saline (PBS), and the decrease in its OECT performance during the soaking experiment was resumed after drying the device by baking (Figures 2B and S5). In our following study, this optimized WNSG formulation was used to make our OECT devices on FPCs.

The mechanical property of the conducting polymer membrane is another essential context for the stability of the OECT device, and we also noticed that mechanical damage may happen during the repeated tests (Figure S6). To protect the device from mechanical damage, we examined two coating materials for the channel: a conductive hydrophobic eutectogel,^36^ and a drop-casted Nafion membrane. Coating with the Nafion membrane enabled hydrophobic sealing of the OECT channel (Figure S7A) without compensation in device performance (Figure 2C). Meanwhile, only a limited OECT response was observed when the device was coated with the eutectogel (Figure S7B). By sealing the PEDOT:PSS membrane with the hydrophobic and ionic conductive material, we not only protect devices from mechanical damage, but also further reduce the water intake of our optimized WNSG formulation-based membrane.

Surprisingly, we found the addition of WPU improved the transient speed of the FPC-OECT devices, and the addition of Nafion further improved the frequency response (Figure 2D), as measured using a voltage divider circuit.^37^ This may be because the addition of hydrophobic compounds reduces the effective volume of the conducting polymer for ion transport and dedoping compared to a channel with the same size as the conventional PEDOT:PSS formulation. Meanwhile, the proton-penetrability of Nafion further promotes ion transport within the channel.^30,31^ The following experiments were based on this stable and robust Nafion membrane-coated WNSG formulation-based membrane.

### DNA-PAAM Hydrogel As a Bioreceptor Module for the OECT-Based Measurement of Aptamer-Small Molecule Interactions

Next, we introduced a bioreceptor module to the developed FPC-based OECT devices for the immobilization of aptamers and the measurement of the small molecule-aptamer interaction. Conventional immobilization methods for bioreceptors in biosensor developments include multiple harsh treatments to the substrate.^38,39^ To realize the immobilization-based aptamer biosensing on OECT, treatment on a separate gate electrode or the specified conducting polymer material providing functional groups for nucleotide immobilization was used by researchers.^40,41^ This adds to the complexity of the device fabrication and limits the flexibility of the design so that only one aptamer sequence can be applied to each device. To achieve evaluation of various aptamer sequences on one OECT device, here, we developed the stand-alone bioreceptor module based on the DNA-polyacrylamide (PAAM) hydrogel. This hydrogel was constructed by a PAAM network grafted with 5′ acrylic phosphoramidite modified single-stranded DNA (acry-ssDNA) (Figure 3A), and it can be prepared by a simple workflow similar to that of the commonly used polyacrylamide gel for electrophoresis. In our system, the acry-ssDNA contains complementary regions to the aptamers; therefore, it acts as the specific capture strand for the aptamers, including a complementary region to it, enabling hybridization-based immobilization of the aptamers (Figure 3A). By simply changing the DNA-PAAM hydrogel module loaded with different aptamer sequences, we can measure the interaction among multiple aptamers and their ligands on the same device without harsh treatment of the substrate. This design gets rid of the time-consuming development of a device for each aptamer, advancing the study of various aptamer-ligand interactions.

**Figure 3:**
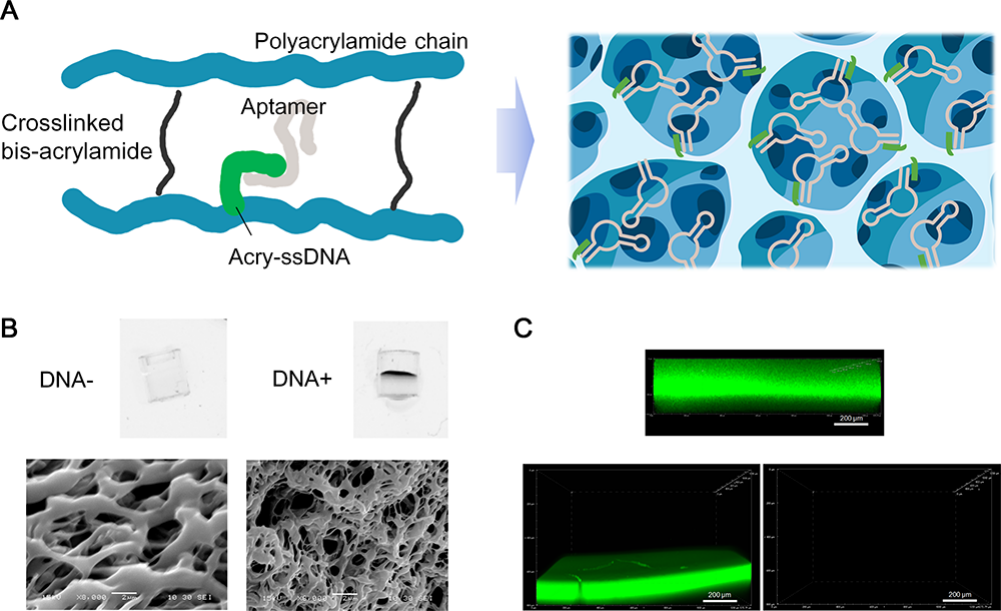
Development and characterization of the DNA-PAAM hydrogel for aptamer immobilization. (A) Illustration of the DNA-PAAM hydrogel with aptamers captured in it. Left: illustration of the PAAM polymer chain cross-linked with the acry-ssDNA that captures aptamer strands; right: illustration of the porous structure of the DNA-PAAM hydrogel with aptamers captured by the cross-linked acry-ssDNA at the surface of the pores. (B) SYBR Gold staining (upper) and scanning electronic microscopy (SEM) of PAAM (DNA−) and DNA-PAAM (DNA+) hydrogels. The SYBR Gold dye was loaded onto the upper surface of the hydrogels. The scale bar in the lower panel is 2 μm. (C) Confocal microscopic photographs of the DNA-PAAM hydrogel. Upper: Hydrogel was stained by SYBR Gold. Lower: DNA-PAAM hydrogel loaded with an aptamer containing a complementary region to the capture strand (left) or a nonspecific strand (right), stained with the double-stranded DNA specific SYBR Green. The concentrations of both strands were 10 μM. The scale bar is 200 μm.

As visualized with a scanning electron microscopy (SEM) (Figure 3B) instrument, the DNA-PAAM hydrogel retained the interconnected microporous structure found in the PAAM hydrogel.^42^ Compared to the PAAM hydrogel, the DNA-PAAM hydrogel of the same concentration of acrylamide displayed a smaller pore size. This may be because the addition of the acry-ssDNA in fact increases the monomer concentration for gelation, which reduces the pore size of the hydrogel.^43^ The presence of the grafted acry-ssDNA in the hydrogel was verified via SYBR Gold staining of the ssDNA strands (Figure 3B) and quantitation of the unbound ssDNA (Table S1). After loading and incubation of the aptamer, an aptamer immobilized layer with a thickness of around 150 μm at the upper surface of the DNA-PAAM was obtained, as observed in confocal microscopy through the staining of the double-stranded regions formed between the acry-ssDNA and the aptamers using the double-stranded DNA specific dye SYBR Green (Figure 3C). To this stage, the bioreceptor module for OECT-based investigation of aptamer-ligand interaction is ready to be used.

After the development of the aptamer-captured DNA-PAAM hydrogel (Apta-gel), we applied it to our FPC-based OECT devices for measurement of the small molecule-aptamer interactions (Figure 4A). The foundation of this idea is that the shift in conformation of target-bound immobilized aptamers may induce an impedance change which can be observed by the nonfaradic, or “capacitive”, electrochemical impedance spectroscopy (EIS).^44^ As a neutral and hydrophobic small molecule, the investigation of cortisol binding to its aptamer requires a high sensitivity of the biosensor. To fully validate the feasibility of our developed system, we chose a recently reported cortisol aptamer,^25^ which was used for cortisol biosensing on an organic field effect transistor. This aptamer will be referred to as “Ref” in the rest of the manuscript, and its sequence can be found in Table S2. We first used the nonfaradic EIS to validate the capacitance change of the immobilized aptamer layer induced by target binding of the ref (Figure S8A). Then we applied the Apta-gel loaded with this aptamer to the FPC-based OECT devices developed in the last section using a polydimethylsiloxane (PDMS) mold and a glass ring-based sample chamber, as illustrated in Figure 4A. The current flow through the microporous hydrogel may undergo two pathways: flowing along the interconnected micropores or flowing across the aptamer immobilized interface into the hydrogel (Figure 4A), which can be described as a resistor and a capacitor, respectively. Hence, an equivalent circuit including a resistor in parallel with a capacitor was used to describe the Apta-gel applied on the OECT (Figure 4A).

**Figure 4:**
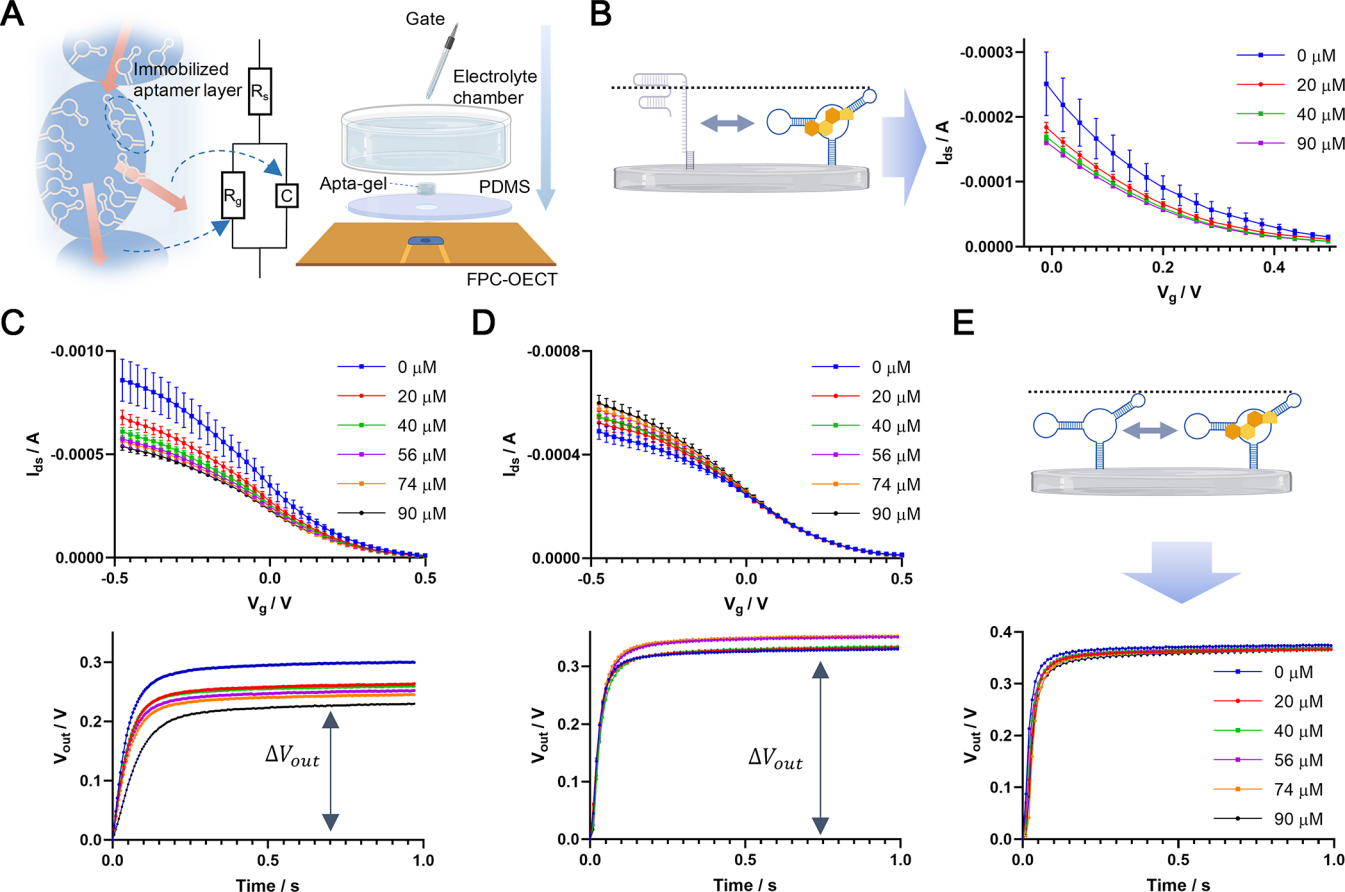
Measurement of small-molecule-aptamer interaction with Apta-gel on FPC-based OECT. (A) Left: illustration of ion flow (red arrows) in the porous Apta-gel and its equivalent circuit. The dashed blue arrows show the corresponding circuit element of the current pathways. Right: Illustration of the integrated device for investigating the interaction between aptamer and its ligand. (B) Shift in the transfer curve of an FPC-OECT induced by ligand-aptamer interaction. The left panel illustrates the interaction that involves an aptamer conformational change. (C) Transfer curve shift and transient response amplitude upon the induction of cortisol at 21 °C. (D) Transfer curve shift and transient response amplitude upon induction of cortisol at 35 °C. The upper and lower panels in (C, D) are two separate experiments using the same OECT setting but taking different signal outputs. Error bars indicate ± S.D., *n* = 3. (E) Transient response of the OECT platform to cortisol induction at 25 °C. The top panel illustrates the ligand-aptamer interaction that does not involve a conformational change. A final concentration of 10 μM for the aptamers was used for the preparation of the Apta-gel.

A shift in the transfer curve of the integrated system was observed upon the addition of cortisol at around 21 °C (Figure 4B). Based on the same aptamer, similar responses were also recorded in control experiments with OECT devices using different aptamer immobilization methodologies, including direct grafting on the OECT channel (Figure S8B) or functionalization on a polyethylene glycol (PEG) hydrogel-based interface via the 1-ethyl-3- (3-(dimethylamino)­propyl) carbodiimide/*N*-hydroxysuccinimide (EDC/NHS) coupling chemistry^45^ Figure S8C). Notably, the shift in transfer curve induced by the addition of cortisol still appeared in the experiment using the same Apta-gel used in Figure 4B after washing in PBS for 12 h at 4 °C (Figure S9A). These results suggested that the same ref aptamer present in the OECT system in different forms and conditions can induce similar responses in the electrochemical output to the cortisol induction.

In later experiments, we compared the response of the Apta-gel loaded OECT to cortisol addition in the transfer curve (upper part of Figure 4C) and the transient curve (lower part of Figure 4C). We noticed that the response amplitude (changes in the output signal *V*_out_, Δ*V*_out_) in the transient curve corresponds to the shift in the transfer curve (Figure 4C). This correlation is because the transient response amplitude reflects the magnitude of change in the device output from an “on” state to an “off” state, which is also recorded in a transfer curve. The drain-source current (*I*_ds_) approaches 0 at high *V*_g_ (i.e., the “off” state) in a transfer curve, so the shifts in the transfer curve during addition of cortisol reflects the change of *I*_ds_ at “on” state of the device (i.e., low or negative *V*_g_), which is the magnitude of the “on” and “off” difference. Hence, the transient response amplitude can represent a shift in the transfer curve. Moreover, the transient response amplitude ΔV_out_ can serve as the output signal for the investigation of the interaction between the aptamer and its ligand. Notably, the DNA-PAAM hydrogel loaded with nonspecific ssDNA probes showed no significant response to the addition of the ligand molecule (Figure S9B) and the transient responses of the Apta-gel loaded system were relatively stable at different temperatures (Figure S9C).

We anticipated that the response of the aptamer-loaded OECT to ligand induction is correlated with the conformational change of the aptamer strands. To verify this idea, we used our platform to measure two aptamers that undergo different binding mechanisms, called CSS.2 and TESS.1, respectively^26^ (Table S2). CSS.2 has a rather stable secondary structure in the absence of its cognate ligand, cortisol, which was supposed to be its binding structure (Figure S9D). OECT loaded with CSS.2 did not show a significant response to the induction of cortisol (Figure S9E). On the other hand, TESS.1 was predicted to show no stable secondary structure in the absence of its cognate ligand, testosterone (Figure S9D), while the formation and stabilization of the G-quadruplex structures during the interaction with its ligand were measured in circular dichroism (CD) spectrum.^26^ Correspondingly, a decrease in the Δ*V*_out_ output was induced in our OECT platform loaded with TESS.1, yielding an apparent affinity lower (higher *K*_D_) than that reported (Figure S9F, Table S3). These results suggest that the response to the aptamer-ligand interaction on our OECT platform indicates a conformational change in the aptamer strands during their interaction with the ligands.

### Aptamer Conformational Change upon Binding Induces the Response in OECT Measurements

Interestingly, opposite responses on our device were observed at higher (35 °C) and lower (21 °C) temperatures for the same aptamer, in both transient and transfer response (Figure 4C,D), while the ligand-free transient responses of the Apta-gel loaded OECT are similar among different temperatures (Figure S9C). To be specific, an increase in Δ*V*_out_ was induced by the addition of cortisol at 35 °C (Figure 4D) while a decrease in that was induced at 21 °C (Figure 4C). Meanwhile, no significant response was recorded at 25 or 30 °C, indicating that the conformation selection binding dominates at this temperature range (Figures 4E and S9G). Such different responses to the same ligand-aptamer interaction are not commonly seen, but the opposite responses of different ligand-aptamer interactions on the same organic transistor device were reported previously, which was presumed as the result of different changes in the height of the immobilized aptamers induced by the ligands.^46^ We hypothesized that the opposite responses on our OECT platform to ligand induction reflect the different conformational changes upon binding of the tested aptamer at various temperatures.

As the shift of the transfer curve also appeared when *V*_g_ ≤ 0 V, which cannot be explained solely by the interface capacitance model,^23^ we considered the model where the aptamer layer on the Apta-gel acts as a charged capacitor introducing an offset to the gate potential.^24,47^ The equivalent circuit of this model is described in Figure 5A, where the equivalent circuit of the Apta-gel as described in Figure 4A is connected in series with the equivalent circuit of the channel.^48–50^ Considering the interconnected microporous structure of the hydrogel, allowing the current flow through the gel-electrolyte interface multiple times, a constant phase element (CPE), instead of an ideal capacitor, would better describe the actual response of the system.^51^ The OECT channel can be described as a resistor in parallel to a Warburg Open (*W*_O_) element,^48–50^ as measured in our EIS experiments (Figure S10). The potential offset *U* is given by the equations in Figure 5A, where *V*_g,eff_ and *V*_g,app_ are the effective and applied gate voltage; n is the number of immobilized DNA molecules; *Q* and *Q*_total_ are the net and total charge of each DNA molecule; *t* is the DNA layer thickness; ε_DNA_ and ε_0_ are the permittivity of DNA and vacuum; and ξ is the charge density parameter.^47,52^ According to these equations, the potential offset *U* is correlated to the DNA layer thickness *t* (i.e., the aptamer layer thickness of our device), and the net charge “*Q*” of the DNA molecules, where *Q* is also related to *t* through the dimensionless charge density parameter ξ.^52^ Note that aptamers are negatively charged ssDNA strands, so *Q* and *U* are both negative. A larger magnitude of the potential offset U indicates a more negative *V*_g,eff_ when *V*_g,app_ equals 0 V, which means a larger magnitude of the OECT output at the “on” state of the device according to the transfer profile of our device (Figure 2B). Hence, the increase in the aptamer layer thickness *t* causes a larger magnitude of *U*, resulting in reinforcement of the Δ*V*_out_, which corresponds to a bigger on/off ratio of the OECT devices. Similarly, a binding-induced decrease in the aptamer layer thickness *t* would lead to a decreasing Δ*V*_out_ output as the on/off ratio is getting smaller. Based on this, we further hypothesized that the increasing Δ*V*_out_ response to cortisol induction (Figure 4D) indicates a larger aptamer layer thickness after the ligand-aptamer interaction, while the decreasing Δ*V*_out_ response (Figure 4C) indicates a smaller aptamer layer thickness after the interaction.

**Figure 5:**
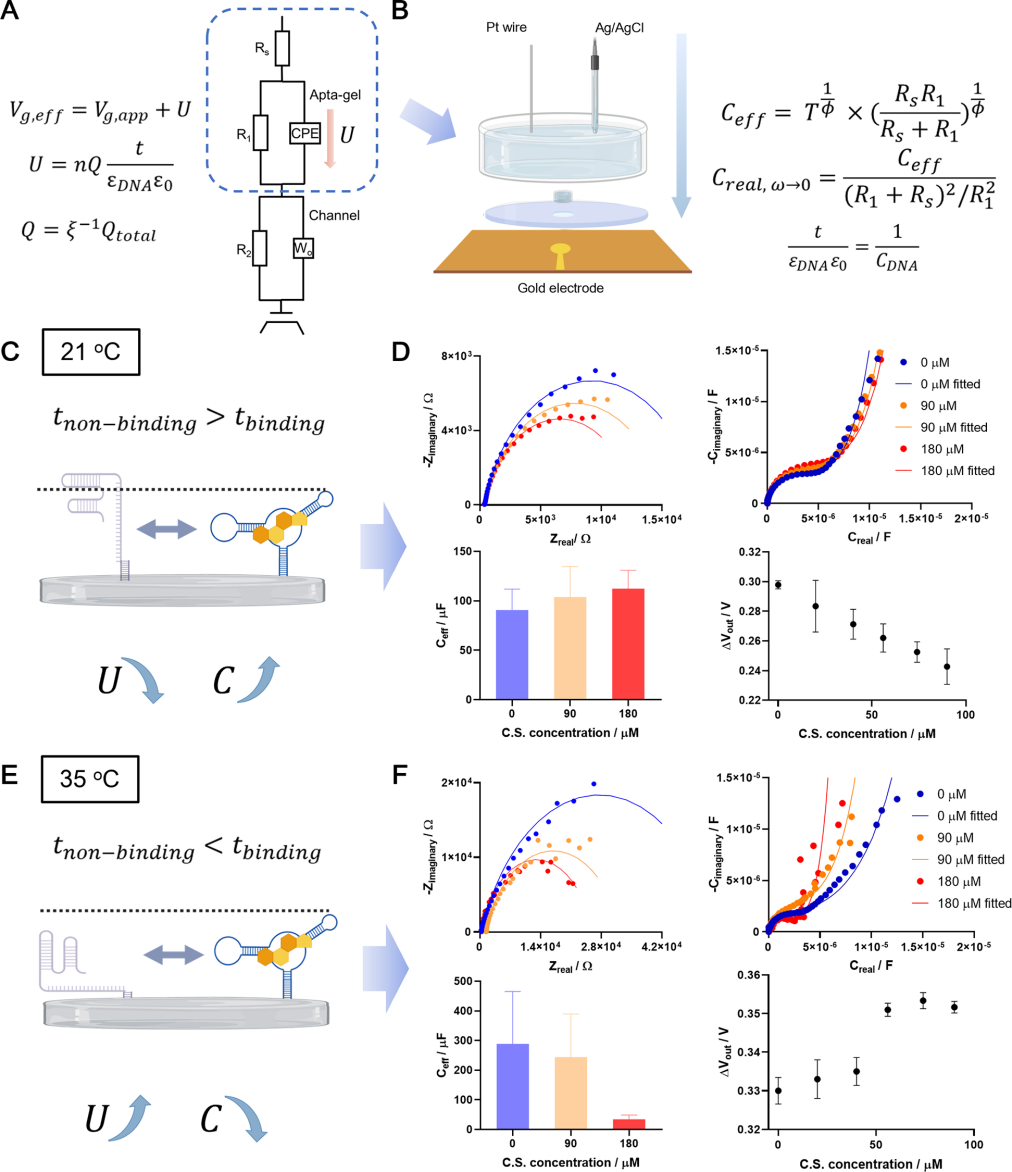
Electrochemical impedance spectroscopy (EIS) of developed Apta-gel upon ligand induction. (A) Equations and equivalent circuit of Apta-gel applied on an OECT. The dashed rectangle highlights the part of the circuit representing the Apta-gel. CPE: constant phase element. (B) Illustration of the EIS setting for measuring Apta-gel and equations for data analysis. (C) Illustration of the decrease in aptamer layer thickness induced by binding. (D) EIS results of Apta-gel upon ligand induction at 21 °C. (E) Illustration of an increase in aptamer layer thickness induced by binding. (F) EIS results of Apta-gel upon ligand induction at 35 °C. The lower right panels of (D) and (F) are the transient response amplitude (Δ*V*_out_) upon ligand induction at the corresponding temperature. Error bars indicate ± S.D., *n* = 3. A final concentration of 10 μM for the aptamers was used for the preparation of the Apta-gel.

We attempted to verify this hypothesis by using nonfaradic EIS to measure the effective capacitance (*C*_eff_) change of the Apta-gel upon ligand induction. The setup of EIS for Apta-gel is shown in the left panel of Figure 5B. As aforementioned, the CPE instead of the ideal capacitor was used to better fit the measured curves (Figure 5A). We considered two capacitance values that are determined by the aptamer layer capacitance *C*_DNA_:*C*_real_,_ω→0_, the real part of the total capacitance as frequency approaching 0 and *C*_eff_, the effective capacitance of the CPE. *C*_eff_ can be derived from the equation listed in Figure 5B, where *T* is the capacitance parameter of CPE, and φ is the constant phase exponent of CPE.^51,53^ Notably, *C*_eff_ is also correlated to *C*_real_,_ω→0._ ^50^ In our previous hypothesis, the reduction in Δ*V*_out_ output at 21 °C was induced by the lessening in aptamer layer thickness *t* (i.e., *t*_nonbinding_ > *t*_binding_), which is expected to cause an increase in the system capacitance in the EIS, according to the equations listed in Figure 5B. In the EIS for Apta-gel, an increase in *C*_eff_ and *C*_real_,_ω→0_ was recorded after the addition of the ligand at 21 °C, agreeing with our hypothesis (Figure 5D). Similarly, in the hypothesis, the raising Δ*V*_out_ upon target induction at 35 °C indicates an increase in aptamer layer thickness (i.e., *t*_nonbinding_ < *t*_binding_), which could induce a dropping system capacitance. We recorded a decrease in *C*_eff_ and *C*_real_,_ω→0_ at 35 °C in EIS after the addition of the ligand (Figure 5E), which was also in agreement with our expectation. Given that no obvious ligand-induced response of the OECT devices can be recorded at 25 and 30 °C (Figures 4E and S9G), even though the affinities of this aptamer to cortisol at these temperatures were measured in ITC (Figure S11), we concluded these results that at temperatures lower than 25 °C the binding induces a decrease in the aptamer layer thickness while at temperatures higher than 30 °C the binding induces an increase in the aptamer layer thickness, and at temperatures between 25 and 30 °C the conformational selection binding takes place. Hence, we considered that the ref aptamer undergoes different binding mechanisms at different temperatures.

In this session, we demonstrated that the presented platform measures the thickness change of the aptamer layer during interaction with its ligand through comparison with the EIS results. We presume that this thickness change is related to the aptamer conformational change during an induce-fit binding. Though we anticipate that the amplitude of the signal change in our OECT-based platform indicates the magnitude of the change in the aptamer layer thickness, we have yet to be able to interpret this correlation quantitatively due to the batch-to-batch variability of the FPC-based OECT devices and the hand-crafted liquid chambers. This issue can be solved by applying our modularized design to more standard and large-scale OECT fabrication methods like screen printing on standardized FPCs.^54^ In this way, a more precise analysis of the correlation in the actual thickness of the aptamer layer can be done. Though we have improved the stability of our devices via optimization of the OECT channel, decay in the device performance is still inevitable after prolonged operation. Background subtracting designs like the wheatstone bridge circuit would be necessary for eliminating the shift in the OECT signal due to decay of the device after repeated measurements,^55^ in cooperation with the low batch-to-batch variation manufacturing of OECT devices.

### Characterization of Cortisol Aptamers in Immobilized Form Using OECT

We then used the developed Apta-gel on FPC-based OECT to characterize the ref aptamer and a novel cortisol aptamer developed in our group called R5MPT. The affinities of both aptamers specific to cortisol were verified with ITC, which is the gold standard in aptamer binding analysis (Figures S11 and S12), and their thermodynamic parameters were obtained based on the ITC results (Figure S13A, Table S3). Overall, the affinity of ref measured by ITC in PBS is close to that of a cortisol aptamer selected with a similar SELEX protocol measured by ITC in a magnesium-free buffer.^56^

First, we tested the apparent affinity of the ref at 18, 21, 35, and 37 °C using the OECT-based platform (Figures 6A and S14), respectively. Corresponding to our previous observations, binding at 18 and 21 °C induced a reducing output signal Δ*V*_out_, while binding at 35 and 37 °C induced an increase in Δ*V*_out_. The amplitude of the signal change (|Δ*V*_out_ – Δ*V*_out,0_|) at 18 and 37 °C was bigger than that at 21 and 35 °C, respectively, so we presumed that more apparent changes were induced in the conformational equilibrium at 18 and 37 °C. This also agrees with the binding heat capacity change (Δ*C*) profile obtained by ITC (Figure 6B), where a small negative binding heat capacity change (−0.1 kcal K^–1^ mol^–1^) between 25 and 30 °C of the ref was recorded. According to the previous studies,^16,17^ such temperature-dependent Δ*C* indicates a temperature dependency of the conformational equilibrium between the binding and nonbinding states (Figure S13B), where a larger magnitude of Δ*C* indicates more significant binding-related conformational change. Hence, this small negative binding heat capacity change indicates that no significant conformational change takes place at this temperature range, agreeing with our observations in the OECT measurements (Figures 4E and S9G). The equilibrium binding affinity (*K*_D_) measured by the OECT device displayed a temperature dependency corresponding to that of the *K*_D_ values measured by ITC (Figure 6C). Besides, the aptamer showed higher *K*_D_ (i.e., lower affinity) in the OECT device than in the ITC. This may be related to the impact of the immobilization of the aptamers.^57^

**Figure 6:**
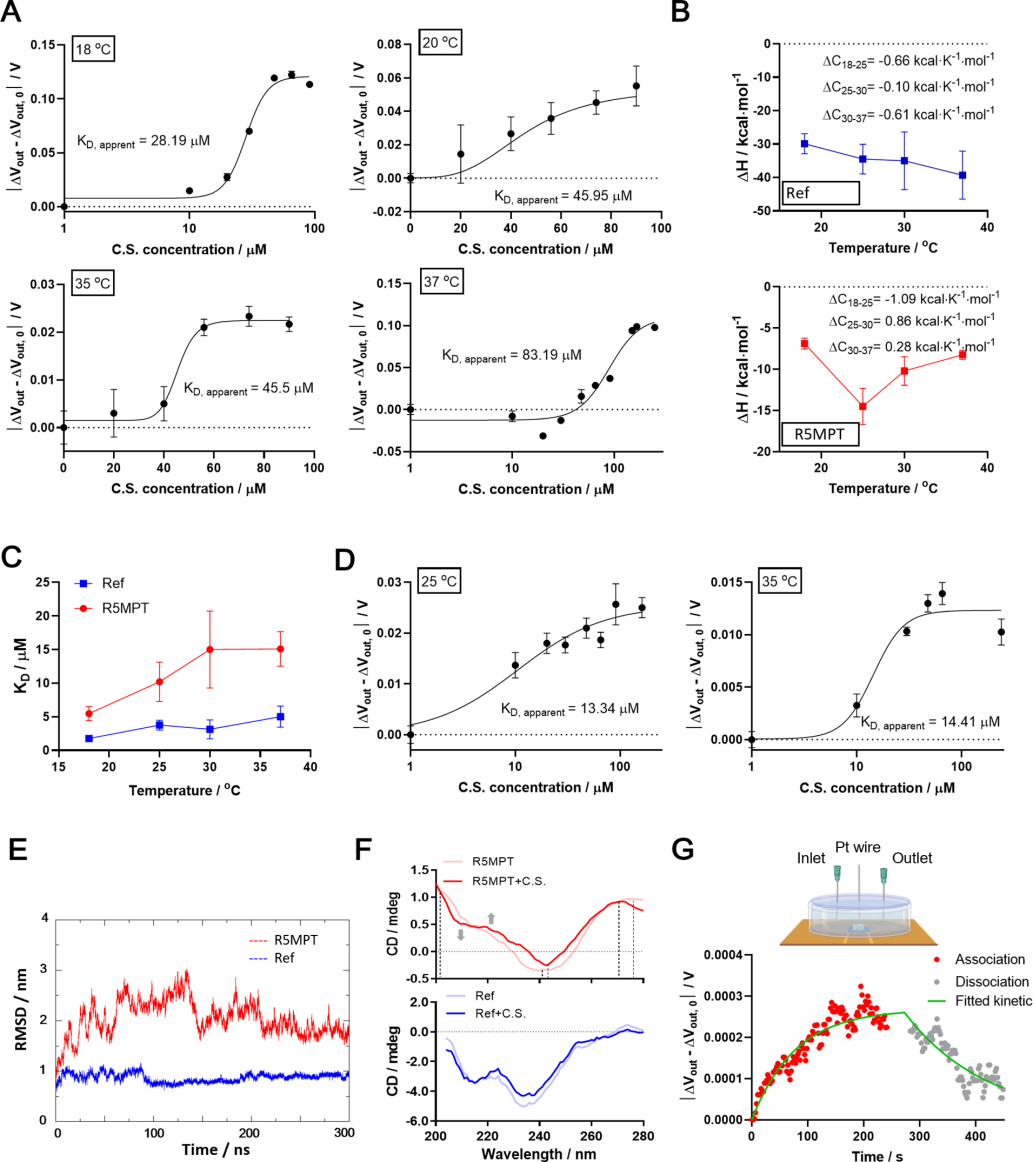
Characterization of two cortisol aptamers with Apta-gel on an FPC-based OECT. (A) equilibrium affinities of ref at different temperatures. (B) Enthalpy and heat capacity change profiles of the ref (upper) and R5MPT (lower) were obtained with ITC. (C) Temperature dependency of K_D_ values of the two aptamers obtained with ITC. Full ITC results can be found in Figures S11 and S12. (D) Equilibrium affinities of R5MPT at different temperatures. (E) Root-mean-square deviation (RMSD) profiles of the two aptamers in molecular dynamic simulation. (F) CD spectra of the two aptamers with (labeled with + C.S.) and without the ligand. The dashed lines highlight the peaks that shifted after the addition of cortisol. The gray arrows highlight the peaks that appeared after ligand addition. (G) Kinetic measurement with Apta-gel on FPC-based OECT. The upper panel showed the illustration of the microfluidic setup. Error bars in A and D indicate ± S.D., *n* = 3. Error bars in parts B and C are the errors calculated by the MicroCal software. A final concentration of 10 μM for the aptamers was used for the preparation of the Apta-gel.

According to the binding-coupled conformational equilibrium model (Figure S13B), before addition of the ligand, aptamer conformations reach an equilibrium in the system consisting of the binding states and the nonbinding states.^16,17^ The presence of the ligand drives the equilibrium in favor of the binding state of conformation. Therefore, a significant shift in the overall conformation can be observed in the case that a nonbinding state is popular in the original equilibrium, which can be described as the “induce-fit mode” of binding, as illustrated in Figure 1. In contrast, if the binding state of the aptamer is intrinsically popular, its binding with ligand may not introduce an obvious change in the equilibrium because the ligand would “select” and bind to the binding state of conformations, which means the “conformation selection mode” of binding takes place (Figure 1). Hence, for aptamers showing temperature-dependent equilibrium of ligand-free conformations, different binding mechanisms may take place at different temperatures, which is the case of the ref aptamer examined. Such temperature dependency of aptamer conformational equilibrium is identical to a temperature dependency of binding heat capacitance,^16,17^ which also corresponds to our ITC results (Figure 6B).

Then we investigated the interaction of a new aptamer (R5MPT) with cortisol using our OECT platform. R5MPT is a cortisol aptamer selected by our group, and its development was stated in detail in another paper under preparation. To begin with, we characterized R5MPT with ITC as a reference (Figure S12, Table S3). This aptamer displayed a significant negative binding heat capacity change between 18 and 25 °C. When the temperature was beyond 25 °C, it showed a positive Δ*C* which diminished with an increasing temperature (lower of Figure 6B). The cortisol binding of this aptamer induced a decrease in Δ*V*_out_ at 25 °C in our OECT platform, showing a binding-related conformational change at this temperature (Figures 6D and S15). As described in previous work,^18^ when the binding event drives the equilibrium from a heterogeneous population of conformations to a certain binding state of conformation, a change in the popular conformations is expected to take place. Similarly, our molecular dynamic results showed that R5MPT displays a heterogeneous population of conformations at 25 °C (Figure 6E). We further verified the conformational change induced by ligand interaction using the circular dichroism (CD) spectrum at the same temperature (Figure 6F). While the ref showed similar profiles in the CD spectrum with and without the ligand (lower of Figure 6F), induction of cortisol led to a shift in the positive peaks at around 274 and 202 nm and the major negative peak at around 240 nm in the CD spectrum of R5MPT (upper of Figure 6F). Notably, the addition of the ligand molecule induced a negative peak at 208 nm in the CD profile of R5MPT (upper part of Figure 6F), indicating a shift in the stem-looplike structure of the B-DNA strand.^58^ Hence, we conclude that compared with ref, R5MPT undergoes a more significant conformational change induced by the binding with its cognate ligand at 25 °C, which induced the response on our OECT platform. Meanwhile, the positive Δ*C* at higher temperatures of R5MPT binding (Figure 6B) indicates exposure of nonpolar groups and dehydration of polar surface during binding, which is usually considered related to the opening of the hydrophobic pocket for binding and the loosening of the ssDNA chain during interaction with the hydrophobic ligands like cortisol.^18,59^ Thus, we expected that ligand binding of this aptamer at a higher temperature like 35 °C leads to a slight increase in the thickness of the aptamer layer, inducing an increasing Δ*V*_out_ but of quite small magnitude. Indeed, the OECT platform obtained an increasing Δ*V*_out_ upon the addition of the ligand with the smallest signal magnitude in this study (Figures 6D and S15). Notably, the *K*_D_ values of R5MPT measured in our OECT platform were close to those from the ITC experiments (Figure 6C,D). Hence, compared to the ref, the affinity of R5MPT to cortisol was less affected by immobilization.

It is worth noting that the conformation of an aptamer in a solution-based system, like in ITC and CD, may be quite different from that in an immobilization-based system,^60^ which is the case of our OECT platform and many application scenarios of aptamers. Hence, further study of how conformational change of aptamer correlates to the thickness of the immobilized aptamer layer would be necessary to fully unleash the ability of this platform for aptamer performance validation. One way to do this could be the molecular dynamics simulation of the immobilized aptamers, considering metastable states of conformations,^9^ which can better describe the correlation between aptamer conformation equilibrium and ligand-aptamer interaction, providing direct conformational information on the aptamers.

Despite the equilibrium affinity, the binding kinetics are another important aspect of the characterization of aptamers. Binders with the same *K*_D_ may display different association and dissociation rates, which has an impact on real applications.^6,13^ Thus, we explored the potential of the Apta-gel and the OECT-based platform in kinetic measurement for aptamer-small molecule interaction. A flow cell was designed for this purpose, as illustrated in the upper panel of Figure 6G. Platinum (Pt) wire was used as the gate electrode instead of the silver chloride electrode (Ag/AgCl) in the flow cell to avoid leakage and simplify the assembly of the device. R5MPT that showed robust response on OECT around room temperature were tested in the kinetic measurement. Though the application of platinum wire led to a lower signal magnitude and larger fluctuations, we still obtained the binding kinetic profile of R5MPT at room temperature (Figure 6G). R5MPT showed relatively slow association (*k*_on_ = 201.4 M^–1^ s^–1^) and slow dissociation (*k*_off_ = 0.0067 s^–1^), yielding a *K*_D_ of 33 μM at 25 °C. The *K*_D_ obtained in the kinetic measurement is in the same order of magnitude as the equilibrium-based measurements at 25 °C.

## Conclusions

In this study, a label-free aptamer characterization platform was developed based on OECTs. This platform is easily accessible thanks to the simple fabrication of the FPC-based OECT devices and the straightforward hybridization-based immobilization of aptamers enabled by the DNA-PAAM hydrogel. The assembled platform can directly measure the conformational change of an aptamer upon interaction with its cognate ligand and provide the apparent affinity of the interaction. It also showed potential in binding kinetic measurements. We successfully used this platform to evaluate a newly selected aptamer from our group by measuring its equilibrium binding affinity and binding kinetics in immobilized settings while investigating its binding-related conformational changes at different temperatures. We believe this provides important information for the application of this aptamer.

Further efforts on improving the batch–batch stability of the device, as mentioned above, and optimization of the microfluidic system would better unleash the potential of this platform for convenient analysis of aptamer binding and the coupled conformational change in both equilibrium and kinetic metrics. In this work, we only considered the neutral small molecule cortisol. In the case of charged molecules, extra signals may be transduced for their interaction with aptamers, due to the accumulation of charge upon the binding of the charged molecules on the bioreceptor layer.^4^ This is good for biosensor design, but it can be an interference to the signal of aptamer conformational change as a characterization platform. Compensation of the extra signal related to the accumulation of the charged ligand molecules would be necessary for the purpose of aptamer characterization, which can be done by quantifying the amount of charge accumulated on the interface.^24^

## Experimental Section

### Experimental Design

This study includes developing a simple fabrication of OECT devices on FPCs, optimization of the channel material of the OECT, development of the bioreceptor module for aptamer binding analysis on the FPC-based OECT, and investigation of two aptamers using the developed DNA hydrogel-based OECT platform. We used EIS, CD, and ITC as reference methods for our newly developed platform. Molecular dynamics simulation was used to understand the equilibrium of the aptamer conformations before binding.

### Fabrication of FPC-Based OECT

Fabrication of OECT on FPC is based on our previous work.^27^ The FPCs are customary and purchased from Shenzhen Yihang Ltd. The channel size was controlled by the distance between the two exposed gold surfaces and their width. The two exposed areas are on two different gold wires insulated in the FPC, with a distance of 0.1 mm between them. The exposed areas are designed to be 0.1 mm × 0.3 mm rectangles, with a distance of 0.13 mm from each other (Figure S4B,C). The FPC is protected by a polyimide tape as a sacrificial layer, and the gold surface for channel fabrication was exposed. The FPC-OECT is fabricated by three steps: spin-coating of the conduction polymer mixture, annealing of the conducting polymer membrane, and peeling of the sacrificial layer. The spin-coating was done with a Laurell spin coater (model WS-650Mz-23NPPB) at 500 rpm for 1 min. The annealing was done by incubating the device on a hot plate at 120 °C for 30 min. The FPCs were treated in O_2_ plasma at 50 W for 1 min prior to spin coating.

### Preparation of Conducting Polymer Mixtures

All of the conducting polymer mixtures used in this work are based on the commercial PEDOT:PSS aqueous dispersion Clevios PH1000 (Heraeus, Germany). All of the other chemicals used were purchased from Sigma-Aldrich unless otherwise specified. The conventional formulation contains PH1000 (0.75 g), D-sorbitol (0.2 g), ethylene glycol (EG) (50 μL), 4-dodecylbenzenesulfonic acid (DBSA) (4 μL), and (3-glycidyloxypropyl)­trimethoxysilane (GOPS) (10 μL). The WPU blended formulation contains PH1000 (0.9 g), WPU 3–501D (Taiwan PU) (12.5 μL), EG (50 μL), and DBSA (4 μL). The Nafion blended formulation is the same as the WPU formulation, except that WPU is substituted by Nafion D520 (Dupont) (45 μL). The optimized WNSG formulation contains PH1000 (0.9 g), D-sorbitol (0.2 g), EG (50 μL), DBSA (4 μL), WPU 3–501D (12.5 μL), Nafion D520, and GOPS (10 μL). All the mixtures were prepared by mixing all the components except GOPS (if applicable) and sonicating for 1 h, followed by the addition of GOPS (if applicable) and another sonication for 15 min.

### Preparation of the Hydrophobic Coating on FPC-Based OECT

For the Nafion membrane coating, 5 μL of Nafion D520 dispersion is directly loaded on each fabricated channel. After incubating at 60 °C for 30 min, the Nafion membrane is formed.

For the eutectogel-coated OECT, the hydrophobic eutectogel was prepared, as described by Li et al.,^36^ directly on the fabricated channel.

### OECT Characterization

Channel resistance was measured by a Fluke 28II true RMS multimeter. OECT characterization was done with a Keithley 2612B sourcemeter (SMU) of Tektronix. Phosphate-buffered saline (PBS) (BupH, Thermo) was used as the electrolyte for OECT operation. For the characterization curve, a screening of drain-source voltage from 0 to −0.8 V was conducted under each gate voltage, respectively. For the transfer curve, screening of gate voltage from 0 to 0.5 V was done with a fixed drain-source voltage of −0.6 V. To obtain a full profile of the OECT response at the cortisol induction experiments in Figure 4C,D, the range of gate voltage screening was expanded to −0.5 to 0.5 V. Transient and frequency response was done with the voltage divider circuit as shown in Figure 2D. The *R*_load_ was set as twice the channel resistance at *V*_g_ = 0 V, as calculated from the transfer curve. *V*_spp_ was set at −1 V, supplied by a Keithley 2612B sourcemeter. *V*_out_ was measured with an MSO7034B oscilloscope (Agilent). For the transient response, a square wave with a frequency of 0.5 Hz, *V*_offset_ = 0.05 V, and *V*_amp_ = 0.1 V was applied to the gate electrode. For the frequency response, a sinusoidal wave of different frequencies was applied to the gate electrode. The gate electrode in this article was a silver chloride electrode, unless otherwise specified. The wave potential was generated with an AFG 31000 function generator (Tektronix).

### Fabrication of the DNA-PAAM Hydrogel Bioreceptor Layer

All of the ssDNA strands, including the aptamer strands and the acry-ssDNA, were purchased from Azenta Life Sciences, Suzhou, China, using the standard 2OD synthesis service with (high performance liquid chromatography) HPLC purification. Their sequences can be found in Table S2. All of the chemicals in this section were purchased from Sigma-Aldrich, unless otherwise specified.

For the DNA-PAAM hydrogel, a gel mixture containing 8% acrylamide, 0.28% bis-acrylamide, and 12.5 μM of acry-DNA is first prepared. After degassing in a sonicator for 10 min, 0.7 μL of 10% ammonium persulfate and 1.4 μL of 5% tetramethylethylenediamine were added to initiate the gelation. The gel forms within 1 h. The gel is then washed with phosphate-buffered saline (PBS) (Thermo Fisher) and stored at 4 °C before use.

For the preparation of the Apta-gel, 30 μL of DNA-PAAM gel was placed in a glass ring with a diameter of 3 mm. Aptamers with the corresponding region to acry-DNA were dissolved in PBS to a final concentration of 10 μM for hybridization-based immobilization on the hydrogel. After the DNA-PAAM gel was washed with PBS, 10 μL of the aptamer solution was directly loaded onto the gel to fully cover the upper surface of the hydrogel. After incubation at 37 °C for 1 h, the gel was washed with PBS and stored at 4 °C before use. For the application of the Apta-gel on the OECT device, a polydimethylsiloxane (PDMS) (Dow Chemical Company) mold for insertion of the hydrogel in a glass ring was designed with an electrolyte chamber made of another glass ring on top of it (Figure 4A).

The cross-linking efficiency of the acry-ssDNA was evaluated by quantifying the released ssDNA in the PBS in the washing step of the prepared gel, using the Qubit 3 Fluorometer with Qubit ssDNA HS quantification kit (Thermo Fisher).

SYBR Gold and SYBR Green (Thermo Fisher) were used to visualize the cross-linked acry-ssDNA and double-stranded region formed between the aptamer and the acry-ssDNA, respectively. Staining was done by loading the 1× dye on top of the prepared hydrogel, followed by an incubation at room temperature for 1 h. Imaging of the stained hydrogel is described in the section “Optical Images and Scanning Electron Microscopy (SEM)”.

### Measurement of Small Molecule-Aptamer Interactions

For equilibrium binding affinity measurements, the OECT setup, as shown in Figure 4A, was used. After assembling the device, transfer curve screening or transient screening is conducted repeatedly until a stable signal is obtained. The introduction of the ligand was done from the lowest concentration to the highest concentration. 15 min incubation is performed after each sample loading, followed by the measurement of the transient or transfer signal.

For kinetic measurements, the OECT setting, as shown in Figure 6G, was used. A flow cell chamber of 100 μL was made by sealing the glass ring-based electrolyte chamber with PDMS. The needles for the inlet and outlet, as well as the platinum wire as the gate electrode, were assembled in the chamber by insertion. A syringe pump (KD scientific, 781100) was used to drive to microfluidic system. To begin with, the assembled chamber containing Apta-gel is filled with PBS at a flow rate of 200 μL/min until a stable transient response is obtained. Then, 30 μM cortisol was flown into the chamber with the same flow rate for association. After flowing the sample for 270 s, PBS is flown into the chamber to initiate the dissociation. The Δ*V*_out_ of each time point was extracted as the signal for further analysis.

### Electrochemical Impedance Spectrum (EIS) Measurements

EIS was done with PalmSens MultiTrace 4. EIS was performed at an AC amplitude of 0.0035 V, a potential of 0.1 V, and a frequency range from 0.1 to 10^5^ Hz. For the EIS with an aptamer immobilized on PEDOT: PSS-coated gold electrode, the immobilization was done as described by Demes et al.,^39^ on a gold electrode spin-coated with the conventional formulation of PEDOT:PSS membrane as mentioned above. For the EIS for Apta-gel, the experimental setting as shown in Figure 4A was used, where the hydrogel is loaded on a gold electrode. In both EIS settings, a silver chloride electrode and a platinum wire were used as the reference electrode and counter electrode, respectively.

### Aptamer Selection

The selection of R5MPT is part of a preparation of the manuscript of our group. In brief, modified capture SELEX based on published methods^26,61,62^ was used to select a predefined library designed for cortisol. The obtained aptamer was truncated to a minimal length that retained its global secondary structure. A sequence corresponding to the acry-DNA was added to the 3′ end of the final sequence for immobilization to the DNA-PAAM gel.

### Isothermal Titration Calorimetry (ITC)

The thermodynamic parameters were measured with a MicroCal PEAQ-ITC (Malvern Panalytical). All the experiments started from titrating 200 μM of cortisol into 20 μM aptamer dissolved in PBS. The injection program, starting with a 0.4-μL preinjection followed by 18 2-μL injections, was used. Control experiments were done as suggested in published methods.^2^ Adjustment of the sample concentration and titration program was done until a stable fitting of the titration curve could be obtained.

### Molecular Dynamics for Aptamer Conformational Equilibrium

DNA aptamer sequences were first converted to RNA by replacing thymine (T) with uracil (U) to generate a high-accuracy 3D model with currently available RNA models. The RNA sequences were processed through trRosettaRNA^63^ to predict their 3D structures, which were subsequently converted back to DNA 3D structures for energy minimization. RMSD representing the amount of metastable states of conformation in the equilibrium was calculated after the molecular dynamics of the sequence on GROMACS.^64^ The ion conditions for simulation were set at 0.005 M MgCl_2_ and 0.1 M NaCl.

### Optical Images and Scanning Electron Microscopy (SEM)

The microscopic images of the hydrogel were taken with a confocal microscope (Nikon C2) with 20× objectives under a 488 nm laser excitation wavelength. Images were analyzed with Nikon NIS-Elements Confocal software. Optical images of the device channels were obtained using the embedded camera of a smartphone (Xiaomi 12X). The SEM images were taken by using the JSM-6060 scanning electron microscope (JEOL). The SEM parameters of acceleration voltage and spot size were set at 15 kV and 50 units, respectively. SYBR Gold staining of the DNA-PAAM hydrogel was visualized via GelDoc Go (Bio-Rad).

### Circular Dichroism (CD) Analysis of the Aptamers and Aptamer-Ligand Complexes

The CD analysis was done with a J-815 CD spectrometer (JASCO). For the analysis of aptamers in the absence of ligand, aptamers were diluted to a final concentration of 1 μM with PBS. For the analysis of aptamer-ligand complexes, cortisol was added to the 1 μM aptamer in PBS to a final concentration of 1 μM. The prepared samples were incubated for 20 min at room temperature before measurement. PBS was used as the background control for the measurement.

### Statistical Analysis

In both equilibrium and kinetic measurements, the normalized output |Δ*V*_out_ – Δ*V*_out,0_| was used for curve fitting, where Δ*V*_out,0_ was the average Δ*V*_out_ in negative measurements. The affinity and kinetic parameters were calculated by curve fitting using GraphPad Prism 10 via the dose–response and binding-kinetic model, respectively. The ITC results were analyzed using MicroCal PEAQ-ITC Analysis Software. The EIS results were analyzed using MultiTrace 4.4 by fitting the data to the corresponding equivalent circuits.

## Supplementary Material



## Notes

Figure S1: Characteristic curve and transfer curve of FPC-based OECT using conventional formulation of PEDOT:PSS membrane; Figure S2: Photographs of devices with different PEDOT:PSS formulations before and after soaking in PSB; Figure S3: Stability of devices using WN and WNG formulations; Figure S4: SEM images of the channel area of fabricated devices; Figure S5: Change transfer curve upon soaking and baking of the device using WNSG formulation; Figure S6: Mechanical damage on the OECT channels after repeated operation; Figure S7: Characterization of the hydrophobic coating for the OECT channel; Figure S8: EIS validation of the ref aptamer and OECT responses of devices using different functionalization techniques; Figure S9: Control experiments for OECT with Apta-gel loaded with different aptamers and at different conditions; Figure S10: EIS measurements of the apta-gel loaded on an FPC-based OECT; Figure S11: ITC profiles of the ref cortisol aptamer; Figure S12: ITC profiles of R5MPT; Figure S13: The binding free energy profile of the two aptamers and the illustration of aptamer conformational equilibrium; Figure S14: Response in transient curves of the reported aptamer upon ligand induction at 18 and 37 °C; Figure S15: Response in transient curves of R5MPT upon ligand induction at 18 and 35 °C; Table S1: Efficiency of acry-DNA cross-linking; Table S2: Sequences in this work; and Table S3: Affinities and thermodynamic parameters of the aptamers used in this work (PDF)

## Abbreviations

CD: circular dichroism
EIS: electrochemical impedance spectrum
FPC: flexible printed circuit
ITC: isothermal titration calorimetry
OECT: organic electrochemical transistor
PEDOT:PSS: poly­(3,4-ethylenedioxythiophene):dextran sulfate
SEM: scanning electron microscope
WPU: waterborne polyurethane.

## References

[1] Dunn M. R., Jimenez R. M., Chaput J. C. (2017). Analysis of Aptamer Discovery and
Technology. Nat. Rev. Chem..

[2] Nucleic Acid Aptamers; Mayer, G. ; Menger, M. M. , Eds.; Springer US: New York, NY, 2023; 2570.

[3] Yu H., Alkhamis O., Canoura J., Liu Y., Xiao Y. (2021). Advances and
Challenges in Small-Molecule DNA Aptamer Isolation, Characterization,
and Sensor Development. Angew. Chem., Int. Ed..

[4] Yoo H., Jo H., Oh S. S. (2020). Detection and beyond: Challenges and Advances in Aptamer-Based
Biosensors. Mater. Adv..

[5] Stangherlin S., Lui N., Lee J. H., Liu J. (2025). Aptamer-Based Biosensors: From SELEX
to Biomedical Diagnostics. TrAC Trends in Analytical
Chemistry.

[6] Froehlich C. E., He J., Haynes C. L. (2023). Investigation of Charged Small Molecule–Aptamer
Interactions with Surface Plasmon Resonance. Anal. Chem..

[7] Stangherlin S., Ding Y., Liu J. (2025). Dissociation Constant
(*K*d) Measurement for Small-Molecule Binding Aptamers:
Homogeneous Assay
Methods and Critical Evaluations. Small Methods.

[8] Daems E., Moro G., Campos R., De Wael K. (2021). Mapping the Gaps in
Chemical Analysis for the Characterisation of Aptamer-Target Interactions. TrAC Trends in Analytical Chemistry.

[9] Rodríguez
Serrano A. F., Hsing I.-M. (2022). Prediction of Aptamer–Small-Molecule
Interactions Using Metastable States from Multiple Independent Molecular
Dynamics Simulations. J. Chem. Inf Model.

[10] Chingarande R. G., Tian K., Kuang Y., Sarangee A., Hou C., Ma E., Ren J., Hawkins S., Kim J., Adelstein R., Chen S., Gillis K. D., Gu L.-Q. (2023). Real-Time Label-Free
Detection of Dynamic Aptamer–Small Molecule Interactions Using
a Nanopore Nucleic Acid Conformational Sensor. Proc. Natl. Acad. Sci. U. S. A..

[11] Onaş A. M., Dascălu C., Raicopol M. D., Pilan L. (2022). Critical Design Factors
for Electrochemical Aptasensors Based on Target-Induced Conformational
Changes: The Case of Small-Molecule Targets. Biosensors (Basel).

[12] Guo S., Lin J., Lin L., Xu W., Guo Y., Xu Z., Lu C., Shi X., Chen L., Yang H. (2023). Selecting Small Molecule
DNA Aptamers with Significant Conformational Changes for Constructing
Transcriptional Switches and Biosensors. Sci.
China Chem..

[13] McKeague M., De Girolamo A., Valenzano S., Pascale M., Ruscito A., Velu R., Frost N. R., Hill K., Smith M., McConnell E. M., DeRosa M. C. (2015). Comprehensive Analytical Comparison
of Strategies Used for Small Molecule Aptamer Evaluation. Anal. Chem..

[14] Ozer A., Pagano J. M., Lis J. T. (2014). New Technologies
Provide Quantum
Changes in the Scale, Speed, and Success of SELEX Methods and Aptamer
Characterization. Mol. Ther Nucleic Acids.

[15] Davlieva M., Donarski J., Wang J., Shamoo Y., Nikonowicz E. P. (2014). Structure
Analysis of Free and Bound States of an RNA Aptamer against Ribosomal
Protein S8 from Bacillus Anthracis. Nucleic
Acids Res..

[16] Bruzzese F. J., Connelly P. R. (1997). Allosteric Properties of Inosine Monophosphate Dehydrogenase
Revealed through the Thermodynamics of Binding of Inosine 5‘-Monophosphate
and Mycophenolic Acid. Temperature Dependent Heat Capacity of Binding
as a Signature of Ligand-Coupled Conformational Equilibria. Biochemistry.

[17] Vega S., Abian O., Velazquez-Campoy A. (2016). On the Link
between Conformational
Changes, Ligand Binding and Heat Capacity. Biochimica
et Biophysica Acta (BBA) - General Subjects.

[18] Sokoloski J. E., Dombrowski S. E., Bevilacqua P. C. (2012). Thermodynamics of Ligand Binding
to a Heterogeneous RNA Population in the Malachite Green Aptamer. Biochemistry.

[19] Plach M., Schubert T. (2019). Biophysical Characterization of Aptamer-Target Interactions. Adv. Biochem. Eng. Biotechnol..

[20] Torricelli F., Adrahtas D. Z., Bao Z., Berggren M., Biscarini F., Bonfiglio A., Bortolotti C. A., Frisbie C. D., Macchia E., Malliaras G. G., McCulloch I., Moser M., Nguyen T.-Q., Owens R. M., Salleo A., Spanu A., Torsi L. (2021). Electrolyte-Gated
Transistors for Enhanced Performance Bioelectronics. Nature Reviews Methods Primers.

[21] Yeung S. Y., Gu X., Tsang C. M., Tsao S. W., Hsing I. (2019). Engineering Organic
Electrochemical Transistor (OECT) to Be Sensitive Cell-Based Biosensor
through Tuning of Channel Area. Sens Actuators
A Phys..

[22] Rivnay J., Ramuz M., Leleux P., Hama A., Huerta M., Owens R. M. (2015). Organic Electrochemical Transistors for Cell-Based
Impedance Sensing. Appl. Phys. Lett..

[23] Nissa J., Janson P., Berggren M., Simon D. T. (2021). The Role of Relative
Capacitances in Impedance Sensing with Organic Electrochemical Transistors. Adv. Electron Mater..

[24] Lin P., Luo X., Hsing I., Yan F. (2011). Organic Electrochemical Transistors
Integrated in Flexible Microfluidic Systems and Used for Label-Free
DNA Sensing. Adv. Mater..

[25] Wang B., Zhao C., Wang Z., Yang K.-A., Cheng X., Liu W., Yu W., Lin S., Zhao Y., Cheung K. M., Lin H., Hojaiji H., Weiss P. S., Stojanović M.
N., Tomiyama A. J., Andrews A. M., Emaminejad S. (2022). Wearable Aptamer-Field-Effect
Transistor Sensing System for Noninvasive Cortisol Monitoring. Sci. Adv..

[26] Yang K.-A., Chun H., Zhang Y., Pecic S., Nakatsuka N., Andrews A. M., Worgall T. S., Stojanovic M. N. (2017). High-Affinity
Nucleic-Acid-Based Receptors for Steroids. ACS
Chem. Biol..

[27] Yeung, S. Y. ; Lin, H. ; Li, Y. ; Fung, C. W. ; Nyein, H. Y. Y. ; Hsing, I.-M. Battery-Powered Wearable Utilizing Flexible Printed Circuit-Based Organic Electrochemical Transistor Embedded with Simple Circuits of Voltage Divider and Regulator for Biosignal Measurement. bioRxiv 2025 10.1101/2025.01.26.630991

[28] Menezes N. P., Nicolini T., Barker M., Mariano A. A., Dartora C. A., Wantz G., Stingelin N., Abbas M., Dautel O. J., Thuau D. (2023). Improved Stability
of Organic Electrochemical Transistor Performance
with a Low Swelling Mixed Conducting Polymer: A Comparative Study
with PEDOT:PSS. J. Mater. Chem. C Mater..

[29] Bießmann L., Kreuzer L. P., Widmann T., Hohn N., Moulin J.-F., Müller-Buschbaum P. (2018). Monitoring
the Swelling Behavior
of PEDOT:PSS Electrodes under High Humidity Conditions. ACS Appl. Mater. Interfaces.

[30] Zhang L., Khayour S., Ren G., He S., Wang J., Yu L., Song Y., Zhu C., Kang X., Zhang Y., Gong Z., Gao K., Wang J., Sheng H., Lu G., Yu H.-D. (2023). Proton-Penetrable
Nafion-Induced Phase Separation in
Organic Semiconductors for High-Performance Organic Electrochemical
Transistors. J. Mater. Chem. C Mater..

[31] Kim Y., Yoo S., Kim J.-H. (2022). Water-Based
Highly Stretchable PEDOT:PSS/Nonionic WPU
Transparent Electrode. Polymers (Basel).

[32] Lee Y. J., Kim Y. H., Lee E. K. (2024). PEDOT:PSS-Based
Prolonged Long-Term
Decay Synaptic OECT with Proton-Permeable Material, Nafion. Macromol. Rapid Commun..

[33] Nardes A. M., Kemerink M., de Kok M. M., Vinken E., Maturova K., Janssen R. A. J. (2008). Conductivity,
Work Function, and Environmental Stability
of PEDOT:PSS Thin Films Treated with Sorbitol. Org. Electron.

[34] Bidinger S. L., Han S., Malliaras G. G., Hasan T. (2022). Highly Stable PEDOT:PSS Electrochemical
Transistors. Appl. Phys. Lett..

[35] He H., Zhang L., Yue S., Yu S., Wei J., Ouyang J. (2021). Enhancement in the Mechanical Stretchability
of PEDOT:PSS
Films by Compounds of Multiple Hydroxyl Groups for Their Application
as Transparent Stretchable Conductors. Macromolecules.

[36] Li M., Liu Z., Hu Y., Li R., Cao Y. (2023). A Hydrophobic Eutectogel
with Excellent Underwater Self-Adhesion, Self-Healing, Transparency,
Stretchability, Ionic Conductivity, and Fully Recyclability. Chemical Engineering Journal.

[37] Braendlein M., Lonjaret T., Leleux P., Badier J., Malliaras G. G. (2017). Voltage
Amplifier Based on Organic Electrochemical Transistor. Adv. Sci..

[38] Meng X., O’Hare D., Ladame S. (2023). Surface Immobilization Strategies
for the Development of Electrochemical Nucleic Acid Sensors. Biosens Bioelectron.

[39] Demes T., Morisot F., Legallais M., Calais A., Pernot E., Pignot-Paintrand I., Ternon C., Stambouli V. (2019). DNA Grafting
on Silicon Nanonets Using an Eco-Friendly Functionalization Process
Based on Epoxy Silane. Mater. Today Proc..

[40] Fenoy G. E., Hasler R., Lorenz C., Movilli J., Marmisollé W.
A., Azzaroni O., Huskens J., Bäuerle P., Knoll W. (2023). Interface Engineering
of “Clickable” Organic Electrochemical
Transistors toward Biosensing Devices. ACS Appl.
Mater. Interfaces.

[41] Diacci C., Burtscher B., Berto M., Ruoko T.-P., Lienemann S., Greco P., Berggren M., Borsari M., Simon D. T., Bortolotti C. A., Biscarini F. (2024). Organic Electrochemical Transistor
Aptasensor for Interleukin-6 Detection. ACS
Appl. Mater. Interfaces.

[42] Wang S., Wang J., Zhu L., Li C., Wu J., Ge S., Yu J. (2024). Aptamer Responsive
DNA Functionalized Hydrogels Electrochemiluminescence
Biosensor for the Detection of Adenosine Triphosphate. Biosens Bioelectron.

[43] Park J., Kim D. (2010). Effect of Polymer Solution Concentration
on the Swelling and Mechanical
Properties of Glycol Chitosan Superporous Hydrogels. J. Appl. Polym. Sci..

[44] Ganguly A., Lin K. C., Muthukumar S., Prasad S. (2021). Autonomous, Real-Time
Monitoring Electrochemical Aptasensor for Circadian Tracking of Cortisol
Hormone in Sub-Microliter Volumes of Passively Eluted Human Sweat. ACS Sens.

[45] Singh N. K., Wang Y., Wen C., Davis B., Wang X., Lee K., Wang Y. (2024). High-Affinity One-Step
Aptamer Selection Using a Non-Fouling
Porous Hydrogel. Nat. Biotechnol..

[46] Nakatsuka N., Yang K.-A., Abendroth J. M., Cheung K. M., Xu X., Yang H., Zhao C., Zhu B., Rim Y. S., Yang Y., Weiss P. S., Stojanović M. N., Andrews A. M. (2018). Aptamer–Field-Effect Transistors Overcome Debye
Length Limitations for Small-Molecule Sensing. Science.

[47] Thompson M., Cheran L.-E., Zhang M., Chacko M., Huo H., Sadeghi S. (2005). Label-Free Detection of Nucleic Acid and Protein Microarrays
by Scanning Kelvin Nanoprobe. Biosens Bioelectron.

[48] Oldenburger M., Bedürftig B., Gruhle A., Grimsmann F., Richter E., Findeisen R., Hintennach A. (2019). Investigation
of the Low Frequency Warburg Impedance of Li-Ion Cells by Frequency
Domain Measurements. J. Energy Storage.

[49] Huang J. (2018). Diffusion
Impedance of Electroactive Materials, Electrolytic Solutions and Porous
Electrodes: Warburg Impedance and Beyond. Electrochim.
Acta.

[50] Itagaki M., Suzuki S., Shitanda I., Watanabe K. (2007). Electrochemical Impedance
and Complex Capacitance to Interpret Electrochemical Capacitor. Electrochemistry.

[51] Lasia A. (2022). The Origin
of the Constant Phase Element. J. Phys. Chem.
Lett..

[52] Rant U., Arinaga K., Fujiwara T., Fujita S., Tornow M., Yokoyama N., Abstreiter G. (2003). Excessive
Counterion Condensation
on Immobilized SsDNA in Solutions of High Ionic Strength. Biophys. J..

[53] Lin C., Ryder L., Probst D., Caplan M., Spano M., LaBelle J. (2017). Feasibility in the
Development of a Multi-Marker Detection
Platform. Biosens Bioelectron.

[54] Zabihipour M., Lassnig R., Strandberg J., Berggren M., Fabiano S., Engquist I., Andersson
Ersman P. (2020). High Yield Manufacturing of Fully
Screen-Printed Organic Electrochemical Transistors. npj Flexible Electronics.

[55] Braendlein M., Pappa A., Ferro M., Lopresti A., Acquaviva C., Mamessier E., Malliaras G. G., Owens R. M. (2017). Lactate Detection
in Tumor Cell Cultures Using Organic Transistor Circuits. Adv. Mater..

[56] Niu C., Ding Y., Zhang C., Liu J. (2022). Comparing Two Cortisol
Aptamers for Label-Free Fluorescent and Colorimetric Biosensors. Sensors & Diagnostics.

[57] Challier L., Miranda-Castro R., Barbe B., Fave C., Limoges B., Peyrin E., Ravelet C., Fiore E., Labbé P., Coche-Guérente L., Ennifar E., Bec G., Dumas P., Mavré F., Noël V. (2016). Multianalytical
Study of the Binding between a Small Chiral Molecule and a DNA Aptamer:
Evidence for Asymmetric Steric Effect upon 3′- versus 5′-End
Sequence Modification. Anal. Chem..

[58] Kypr J., Kejnovska I., Renciuk D., Vorlickova M. (2009). Circular Dichroism
and Conformational Polymorphism of DNA. Nucleic
Acids Res..

[59] Lin P.-H., Yen S.-L., Lin M.-S., Chang Y., Louis S. R., Higuchi A., Chen W.-Y. (2008). Microcalorimetrics Studies of the
Thermodynamics and Binding Mechanism between l-Tyrosinamide and Aptamer. J. Phys. Chem. B.

[60] Rao A. N., Grainger D. W. (2014). Biophysical Properties of Nucleic Acids at Surfaces
Relevant to Microarray Performance. Biomater.
Sci..

[61] Martin J. A., Chávez J. L., Chushak Y., Chapleau R. R., Hagen J., Kelley-Loughnane N. (2014). Tunable Stringency Aptamer Selection and Gold Nanoparticle
Assay for Detection of Cortisol. Anal Bioanal
Chem..

[62] Yang K.-A., Pei R., Stojanovic M. N. (2016). In Vitro
Selection and Amplification
Protocols for Isolation of Aptameric Sensors for Small Molecules. Methods.

[63] Wang W., Feng C., Han R., Wang Z., Ye L., Du Z., Wei H., Zhang F., Peng Z., Yang J. (2023). TrRosettaRNA:
Automated Prediction of RNA 3D Structure with Transformer Network. Nat. Commun..

[64] Abraham, M. ; Alekseenko, A. ; Basov, V. ; Bergh, C. ; Briand, E. ; Brown, A. ; Doijade, M. ; Fiorin, G. ; Fleischmann, S. ; Gorelov, S. ; Gouaillardet, G. ; Grey, A. ; Irrgang, M. E. ; Jalalypour, F. ; Jordan, J. ; Kutzner, C. ; Lemkul, J. A. ; Lundborg, M. ; Merz, P. ; Miletic, V. ; Morozov, D. ; Nabet, J. ; Pall, S. ; Pasquadibisceglie, A. ; Pellegrino, M. ; Santuz, H. ; Schulz, R. ; Shugaeva, T. ; Shvetsov, A. ; Villa, A. ; Wingbermuehle, S. ; Hess, B. ; Lindahl, E. GROMACS 2024.0 Manual; Zenodo, 2024.

