# Supplementary material for: DNA Hydrogel-Interfaced Organic Electrochemical Transistor for the Investigation of Binding-Induced Conformational Change of Small Molecule Aptamers
Source: ACS Appl Mater Interfaces. 2025 Sep 8;17(37):51723–36. doi: 10.1021/acsami.5c11113 (PMC12447391; doi:10.1021/acsami.5c11113)
Supplement: Supplementary file 1 [file am5c11113_si_001.pdf]

Supplementary Information

DNA hydrogel-interfaced organic electrochemical transistor (OECT) for investigation of binding-induced conformational change of small molecule aptamers

Haosi Lin[1], Zibin Zhao[1], Xianzhen Feng[1], Sin Yu Yeung[1], I-Ming Hsing[1]*

[1]Department of Chemical and Biological Engineering, The Hong Kong University of Science and Technology, Hong Kong, China.

**Corresponding author. I-Ming Hsing, Email: kehsing@ust.hk*

This file includes:

Figs. S1 to S15
Tables S1 to S3

Fig. S1.

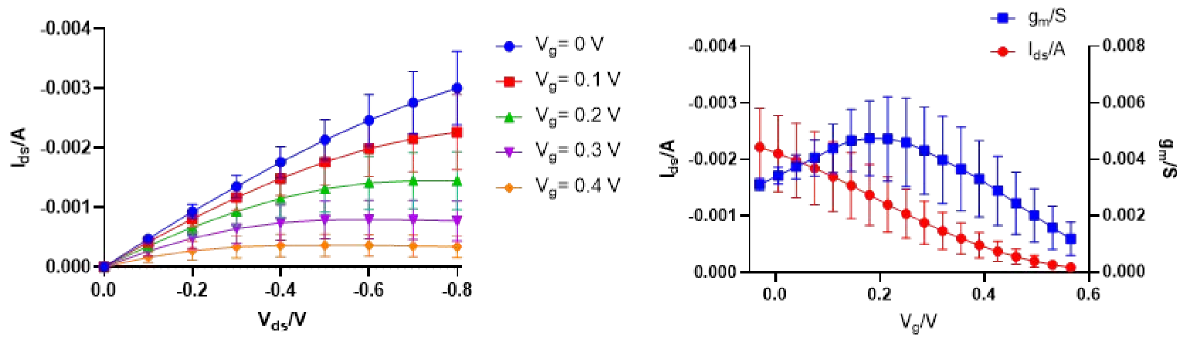

Characteristic curve (left) and transfer curve (right) of FPC-based OECT using conventional formulation of PEDOT:PSS membrane. Error bars indicate S.D., n=2. Channel resistance of this device increased significantly after 3 operations and lost the OECT response afterwards. The transient and frequency responses of this device can be found in Fig. 2C.

Fig. S2.

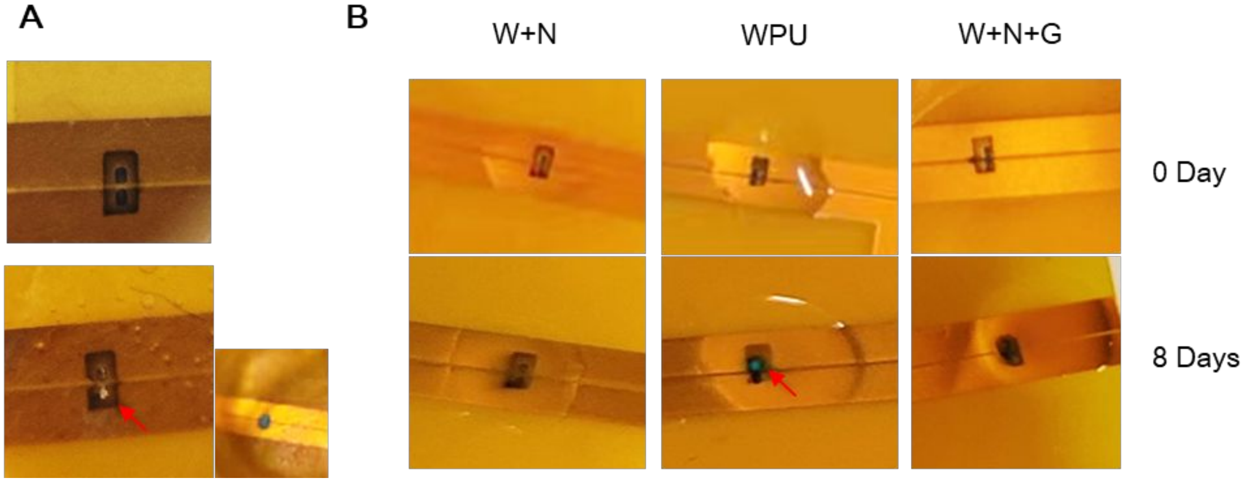

(A) Upper: A newly fabricated PEDOT:PSS membrane of the conventional formulation. Lower: Damaged PEDOT:PSS membrane. The red arrow highlighted the point where the conducting polymer membrane detached from the gold surface. Lower right corner: a rarer case of damage of the membrane appears after continuous contact with aqueous solution where the channel material swelled into a cyan color and became disconnected. (B) 8-day PBS soaking experiment of the modified formulation of PEDOT:PSS membrane. WPU: WPU blended PEDOT:PSS membrane; W+N: WPU and Nafion blended; W+N+G: WPU, Nafion and GOPS blended. The red arrow highlights the point of swelling in a disconnected channel. Notably, though no obvious change in morphology was observed in W+N and W+N+G devices, their resistance increased from 1.6 and 1.2 kΩ to 30 and 130 kΩ, respectively which led to very limited OECT response.

Fig. S3.

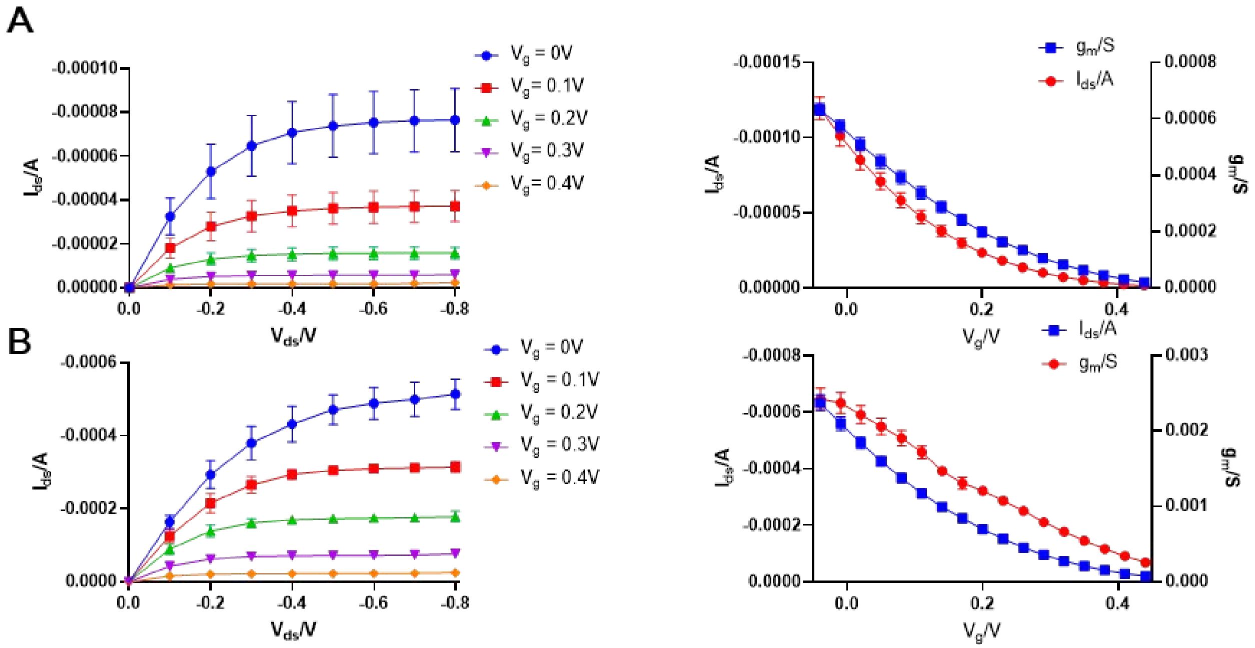

(A) 5-day stability of WPU and Nafion added PEDOT:PSS membrane upon repeated operation. (B) 3-day stability of WPU, Nafion and GOPS added formulation. Introduction of the additives also shifted the transconductance peak to the left, allowing high signal amplification efficiency at low gate voltages.

Fig. S4.

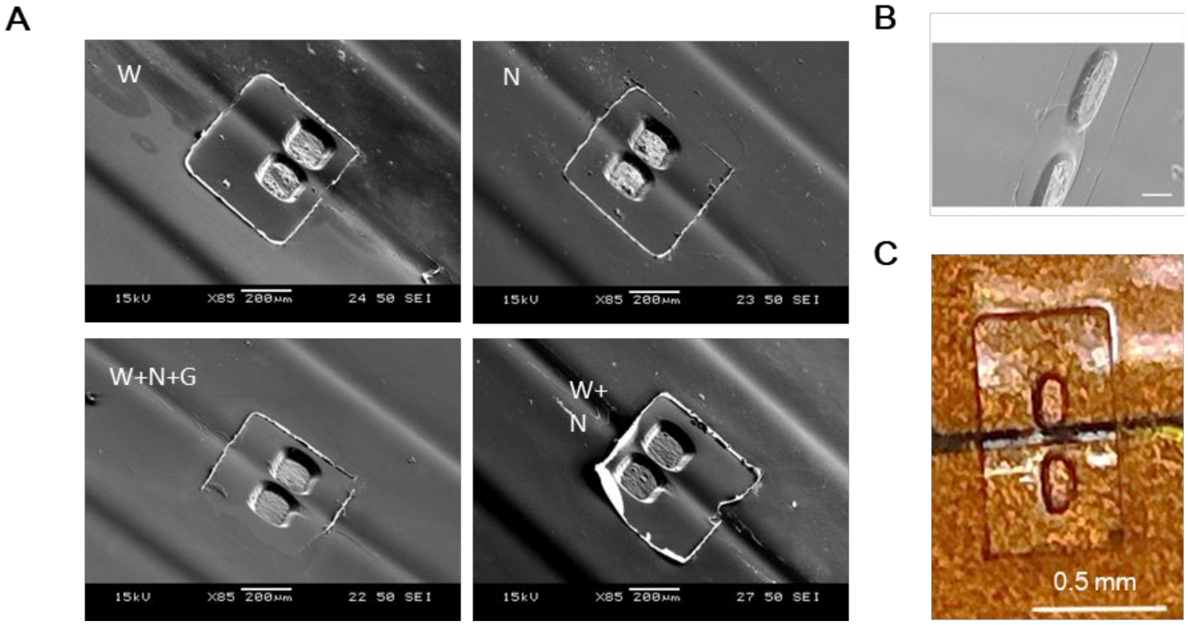

(A) SEM images of the fabricated channels with different formulations. The edges of the conducting polymer membrane showed a trend of peeling off due to the peel-off operation of the sacrificial polyimide tape. The formulation with GOPS (W+N+G) showed less trend of peeling off than the others. (B) The channel area of the fabricated FPC before spin-coating of the PEDOT:PSS membrane. (C) The optical photograph of the channel area of the FPC before spin-coating and covered with the polyimide tape sacrificial layer.

Fig. S5.

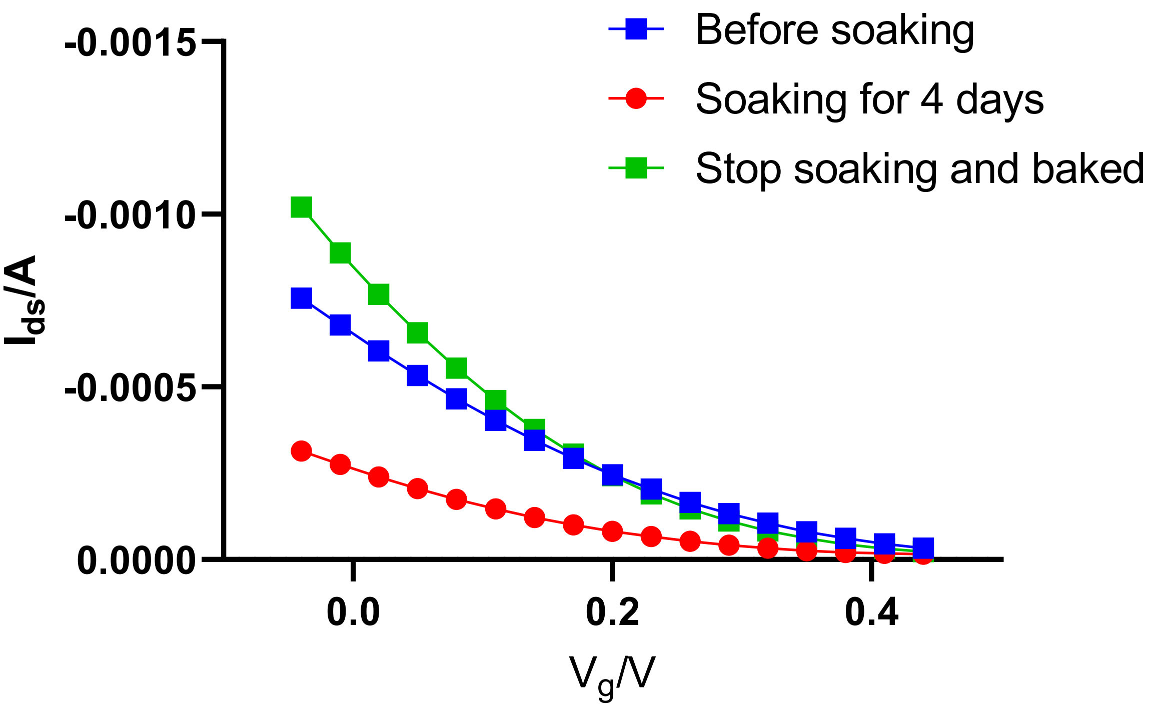

Change transfer curve upon soaking and baking of the optimized PEDOT:PSS formulation (WNSG). This experiment is done simultaneously on the same device with the experiment in Figure 2B. Baking resumed the increase in resistance and the shift in transfer curve due to soaking.

Fig. S6.

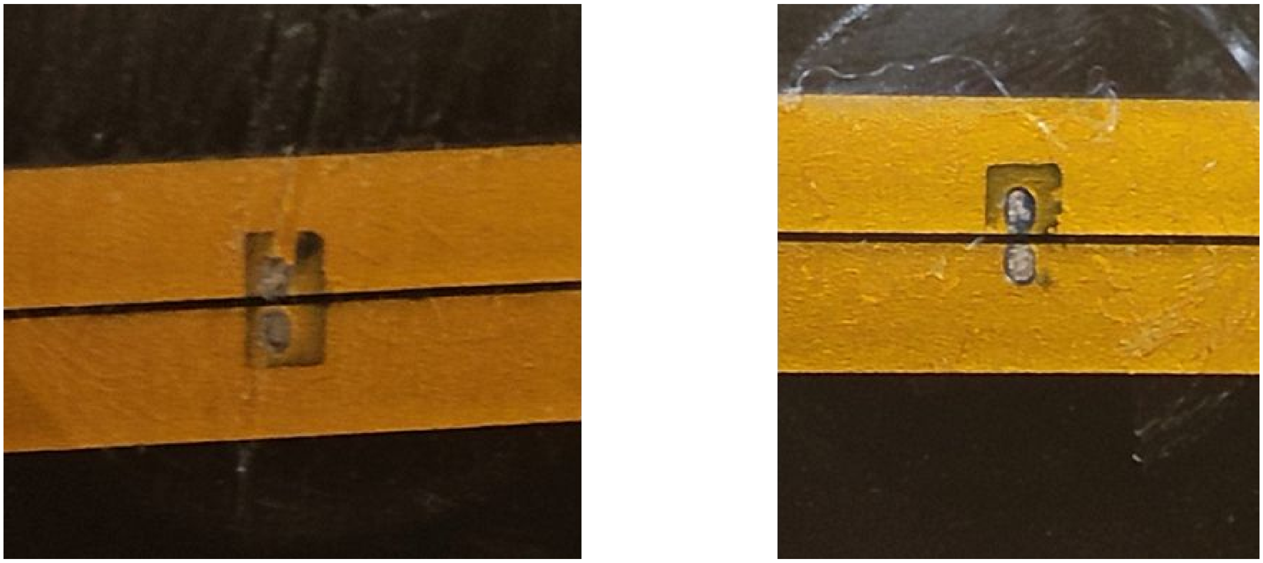

Mechanical damage on the OECT channels after repeated operation. Scratches appeared and a trend of detaching was observed, due to the disassembly and assembly of the electrolyte chamber of the OECT and the hydrogel-based bioreceptor module. In an extreme case (right), half of the conducting polymer was detached from the FPC.

Fig. S7.

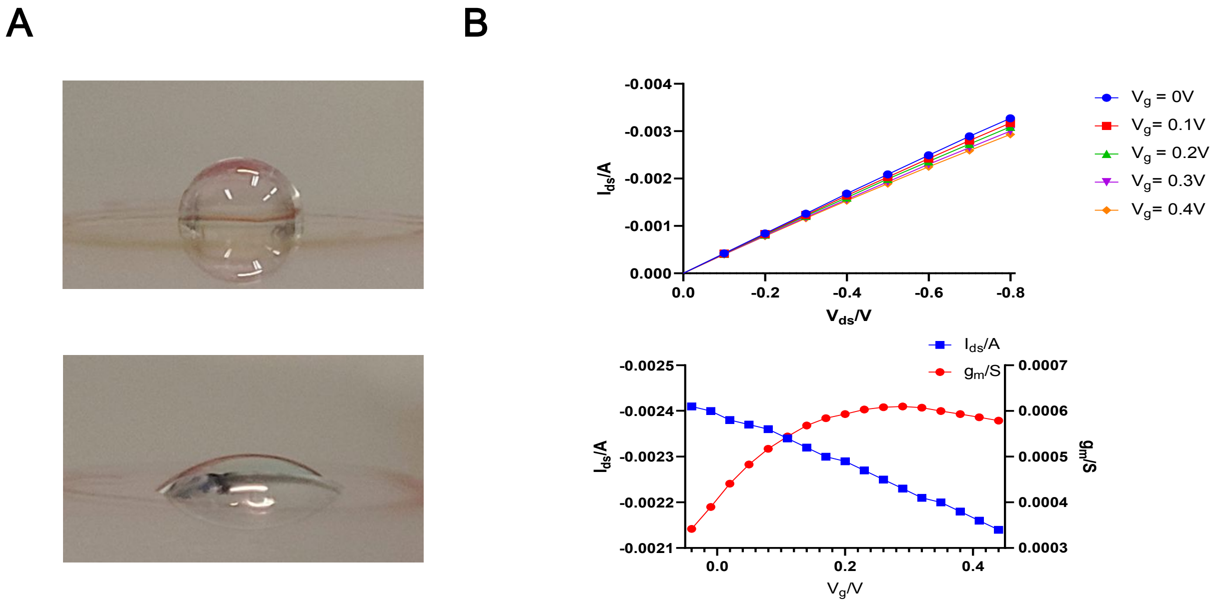

(A) Hydrophobicity of a drop cast Nafion membrane (upper) on glass substrate compared with that of the original glass substrate (lower). (B) Characterization (upper) and transfer curve (lower) of an FPC-based OECT with its channel coated with the hydrophobic eutectogel. Compared to the bare devices, the transconductance of the gel-coated device decreased for one order of magnitude, though of "on" state current retained in the same level of that of the bare devices. WNSG formulation was used for the gel-coated device.

Fig. S8.

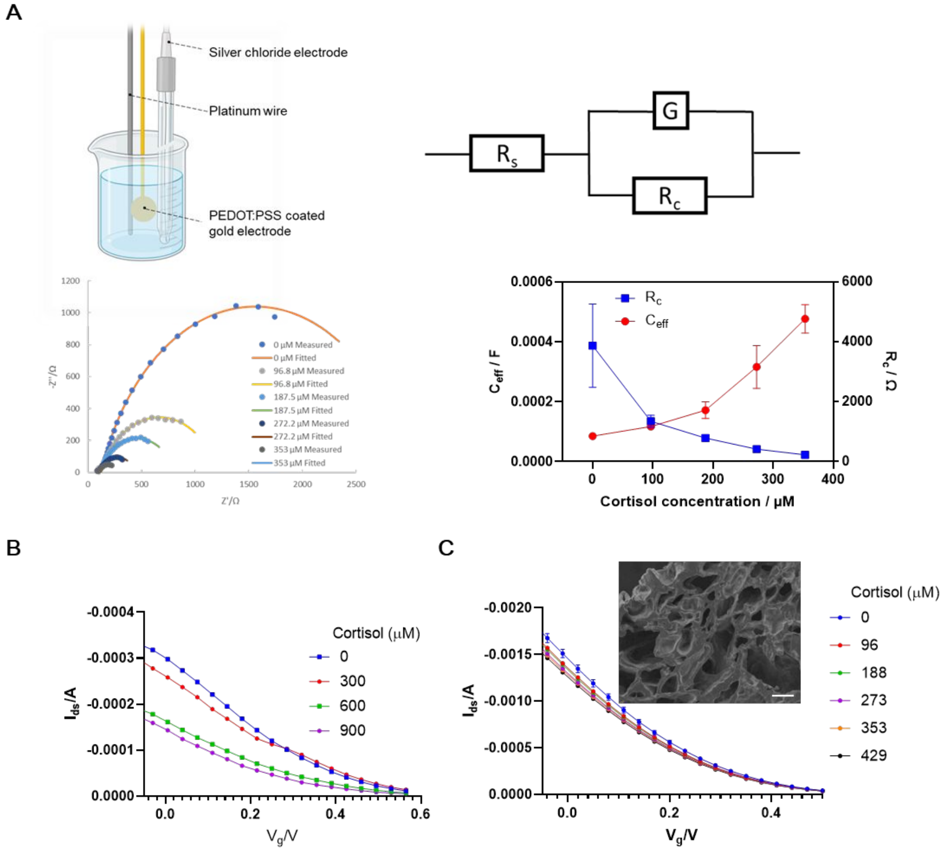

(A) EIS validation of the Ref aptamer binding cortisol. Upper: EIS setting (left) and equivalent circuit (right) for curve fitting. Lower: Nyquist plot of the EIS measurement (left) and the extracted R_c and C_{eff} upon addition of cortisol. The R_c and C_{eff} were calculated using the equation listed in Figure 4B. Error bars indicate S.D., n=3. G, constant phase element. (B) Ligand induced response on a OECT with the Ref aptamer grafted onto the surface of the channel material. This device used the conventional formulation of the PEDOT:PSS mixture to facilitate immobilization via the protocol same as for the EIS measurement. (C) OECT response of the aptamer-ligand interaction of the Ref aptamer immobilized on a porous PEG hydrogel. Error bars indicate S.D., n=3. The inset shows the SEM image of the PEG hydrogel. Scale bar indicates 0.1 mm. The PEG hydrogel contains 10% (v/v) PEGMA, 0.2% (v/v) PEGDA and 1% (v/v) acrylic acid in 1×PBS with pH adjusted to 7.5. Gelation was initiated by the addition of 0.2% (v/v) TEMED and 4% (w/v) APS. The porous structure was realized by cryogelation at -20°C for 12 hours followed by thawing at room temperature. Immobilization of aptamer strands was done by the standard EDC/NHS protocol using amine-labelled aptamer.

Fig. S9.

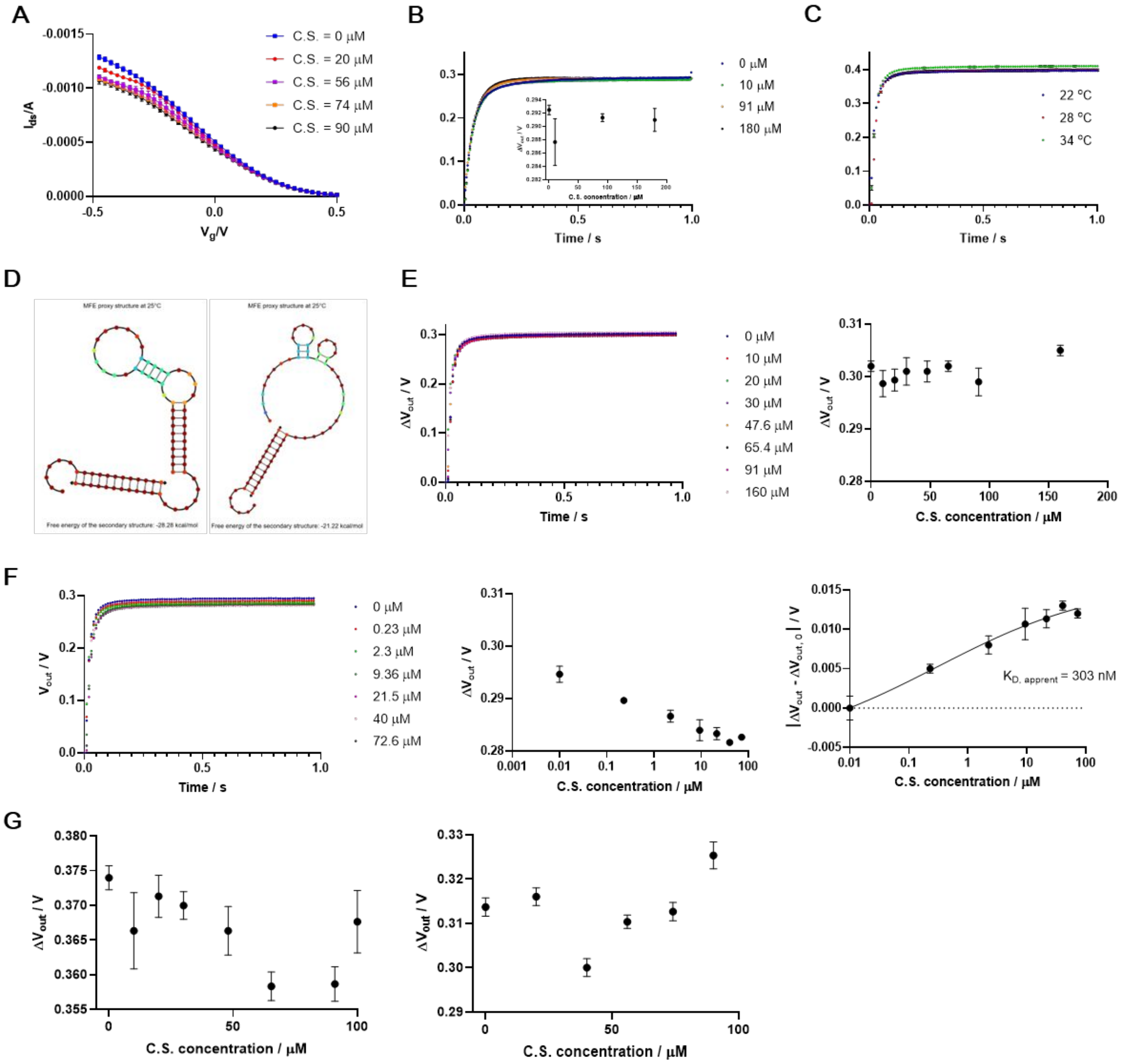

(A) Transfer curve shift of an OECT with a used apta-gel upon ligand induction. This Apta-gel is the same gel used in Figure 4C and was incubated with fresh PBS at 4 ^{o}C for 12h to release the cortisol bond to the aptamer immobilized on the gel before the test in this figure. (B) Transient responses of OECT loaded with DNA-PAAM gel with non-specific ssDNA at different cortisol concentrations. (C) Transient responses of a same Apta-gel loaded with the Ref aptamer at different temperatures without the ligand. (D) Secondary structures of CSS.2 and TESS.1 predicted with NUPACK (nupack.org) (E) OECT response to CSS.2-cortisol interaction. (F) OECT response to TESS.1-testosterone interaction. The calculated $K_{D,\ apparent}$ of 303 nM was higher than the reported K_D of 80 nM measured using a fluorescence-based competitive assay. (G) The response of the Ref aptamer immobilized on OECT to cortisol induction at 25 ^{o}C (left) and 30 ^{o}C (right). Error bars indicate S.D., n=3. No significant trend can be observed from the fluctuation in the output signals.

Fig. S10.

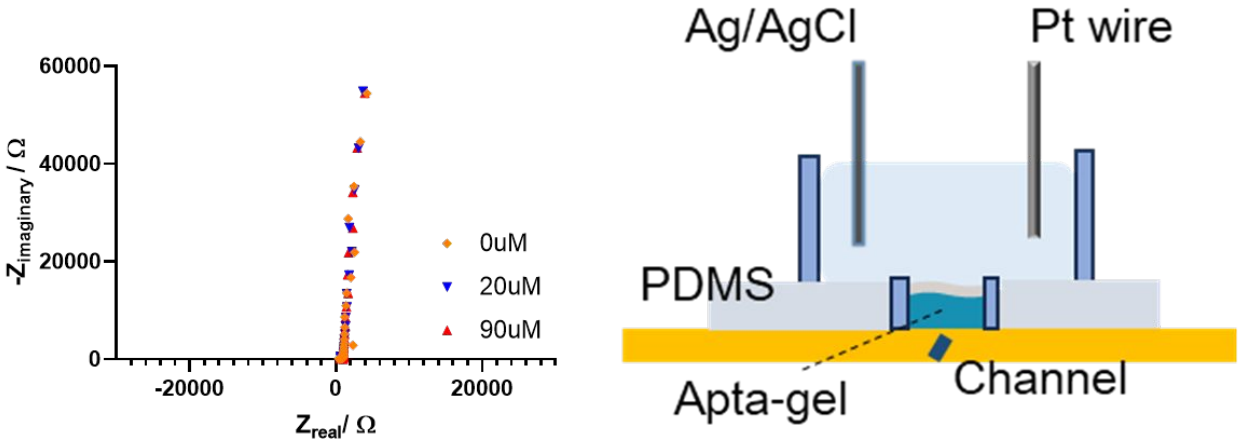

EIS measurements of the apta-gel loaded on an FPC-based OECT. The right panel illustrates the electrochemical setting for the EIS experiment. The drain and source electrode of the channel were short circuited and together act as the working electrode in the EIS set up. The Nyquist plot was mainly determined by the properties of the conducting polymer membrane.

Fig. S11.

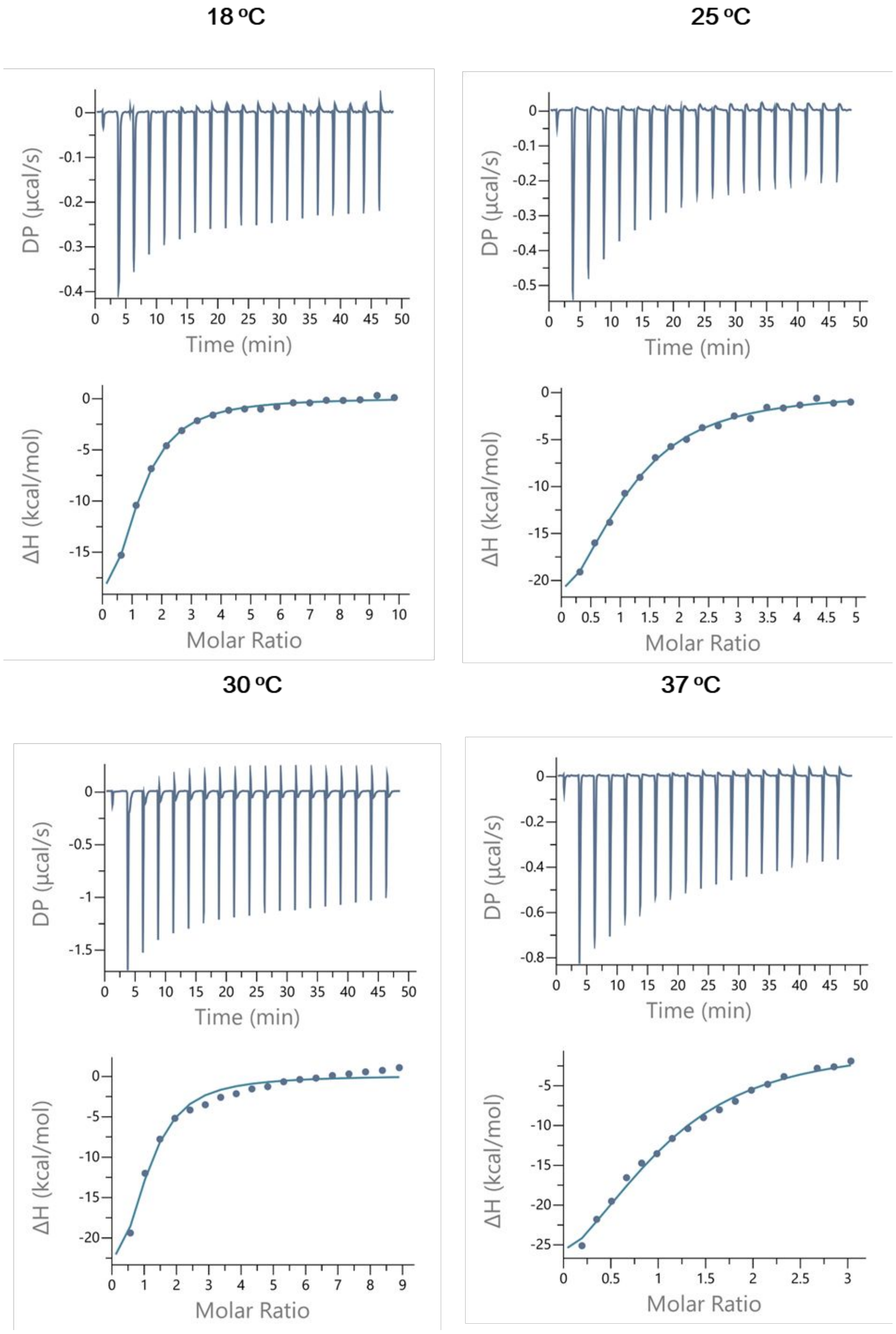

ITC profiles of the Ref cortisol aptamer.

Fig. S12.

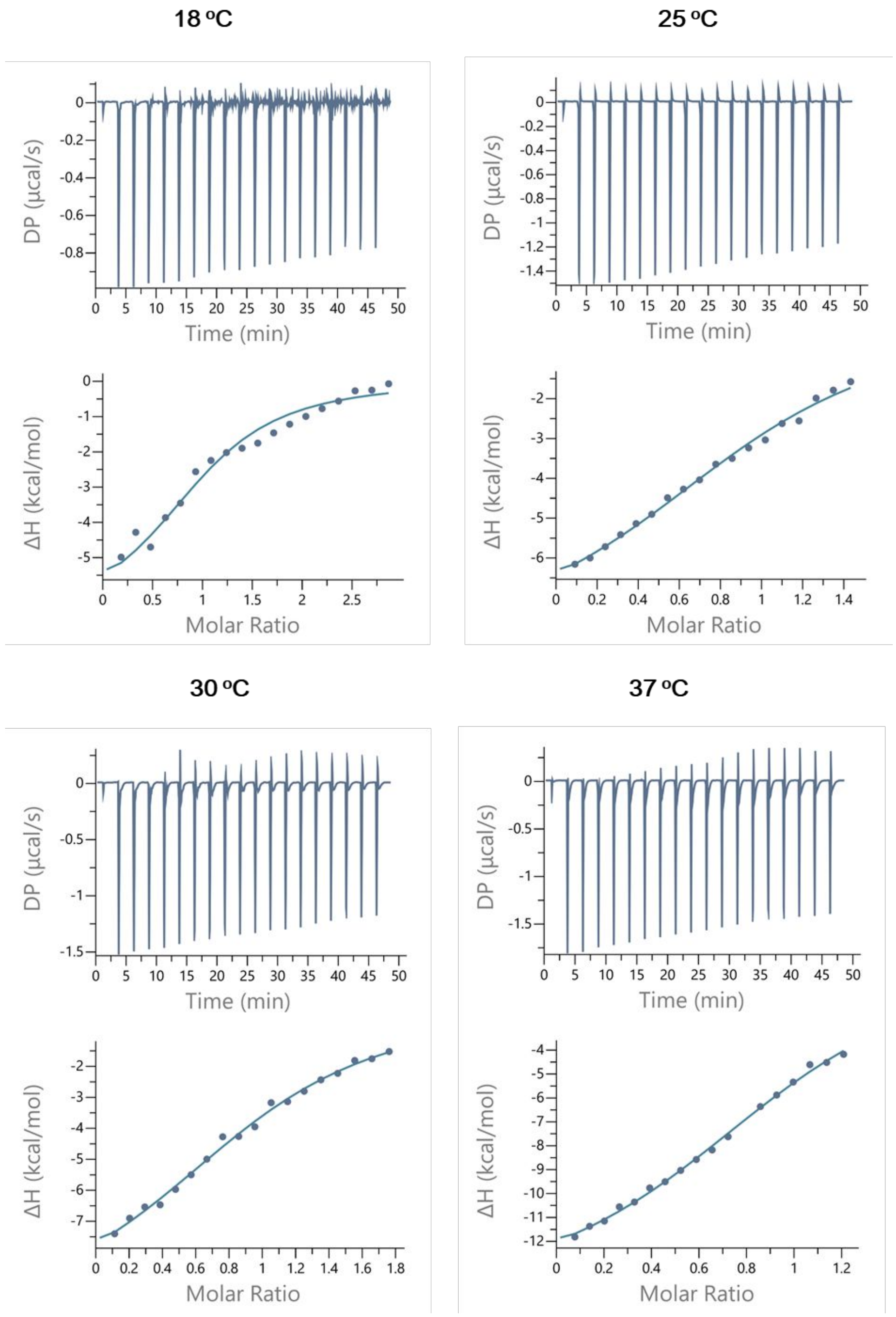

ITC profiles of R5MPT.

Fig. S13.

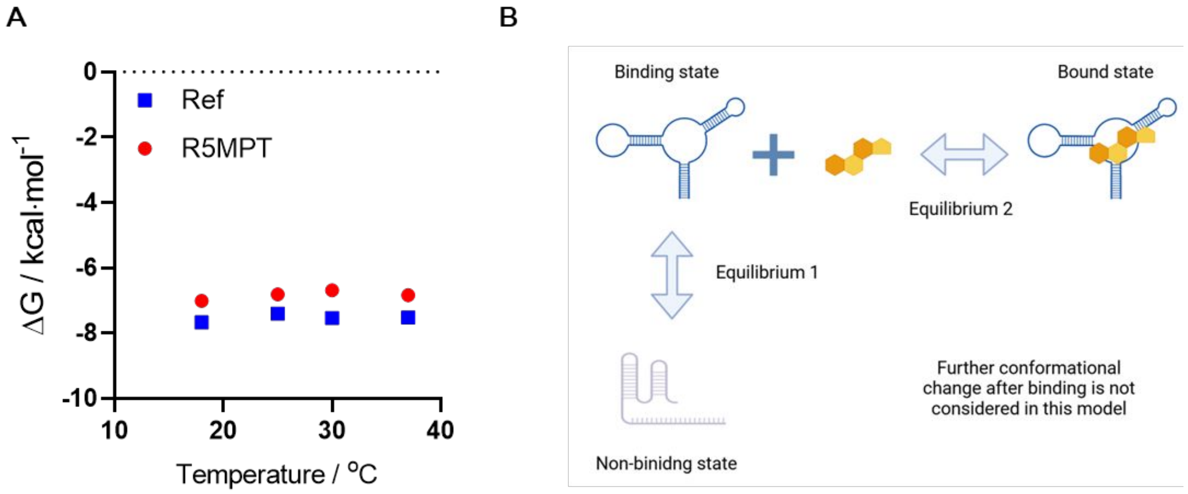

(A) The binding free energy profile of the two aptamers. The binding free energy is not expected to change significantly at different temperatures. (B) Illustration of the equilibrium among ligand, binding state of aptamer and the non-binding state of aptamer. The bound state of aptamer is considered same as the binding state of aptamer. Note that multiple non-binding states may exist simultaneously in the same equilibrium, though only one non-binding conformation is illustrated.

Fig. S14.

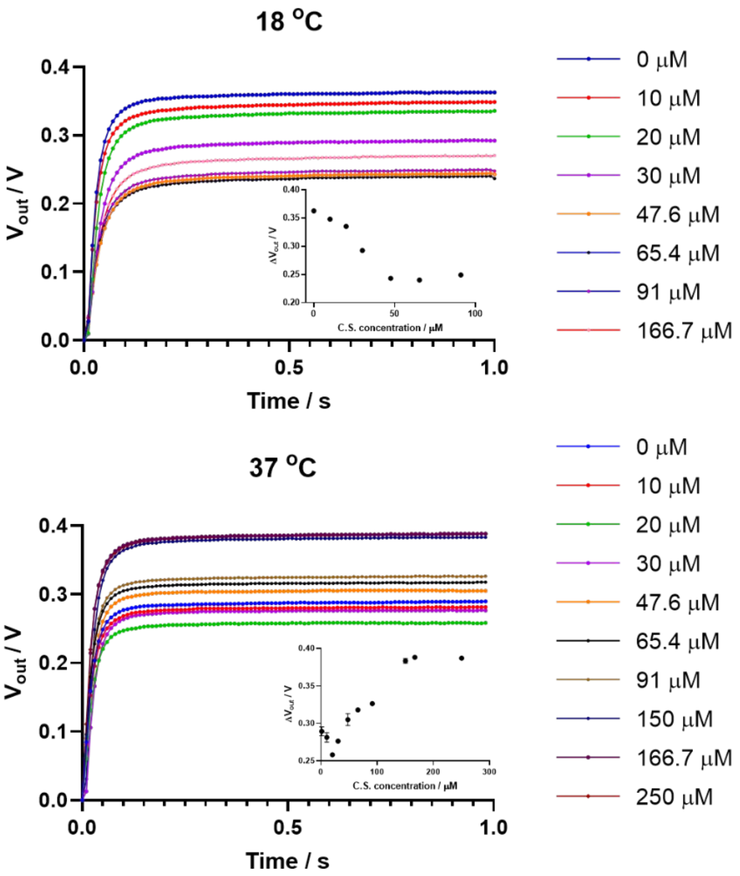

The response in transient curves of the reported aptamer upon ligand induction at 18 °C and 37 °C. The inset showed the extracted ΔV_{out} value under different ligand concentrations. The response of this aptamer at 21 °C and 35 °C can be found in Figure 3C, D and Figure 4B, C.

Fig. S15.

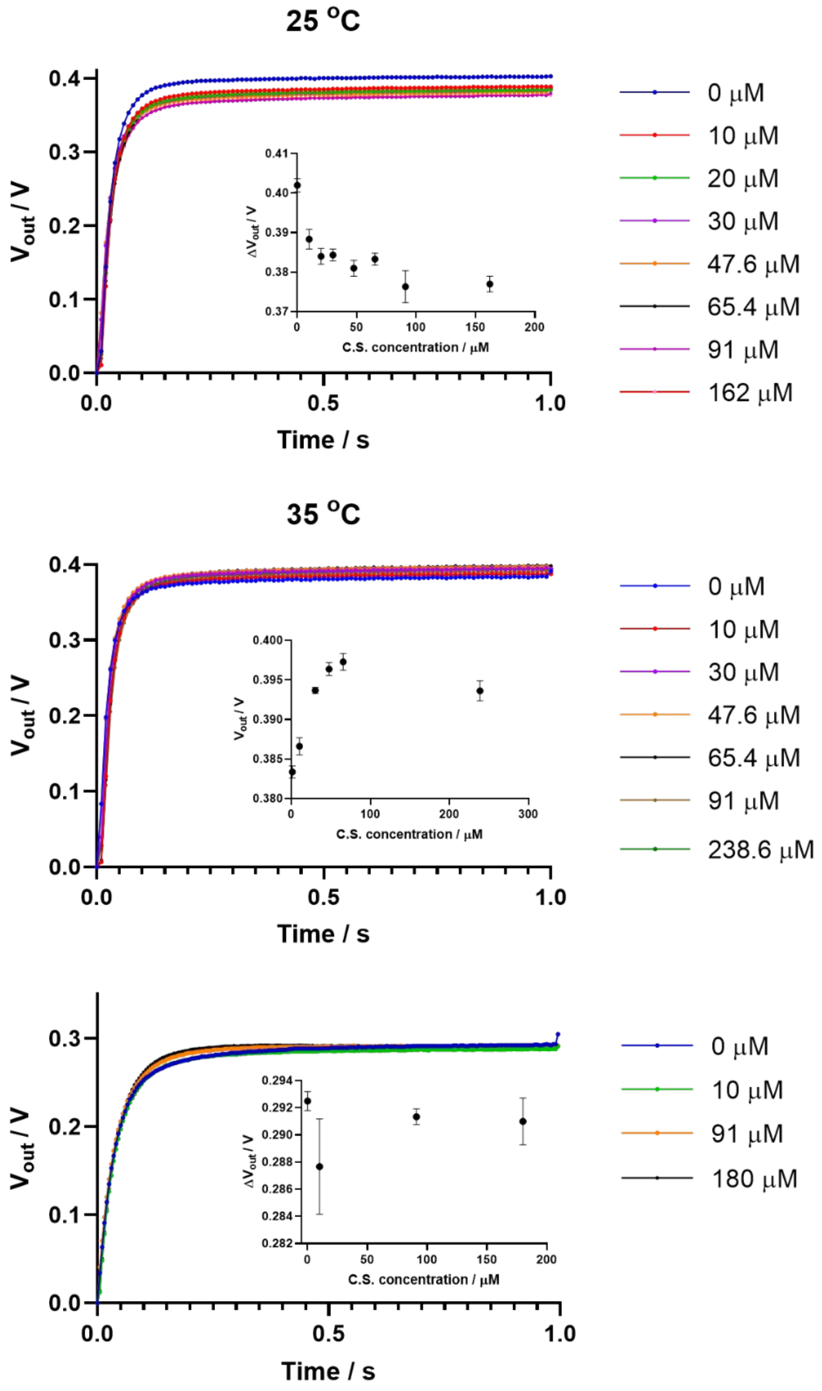

The response in transient curves of R5MPT upon ligand induction at 18 and 35 °C. The insets in each plot show the extracted ΔV_{out} value under different ligand concentrations. The lowest panel shows the response of a DNA-PAAM gel that was not loaded with a target-specific aptamer.

Table S1.

**PAAM concentration**	**Input ssDNA**	**Extracted ssDNA***
4%	300 pmol	72.8 pmol
8%[+]	300 pmol	24.2 pmol

* The crosslinked gel was incubated in PBS overnight, and the ssDNA amount in the PBS was measured with Qubit ssDNA kit.
[+] 8% PAAM was used in the rest of this manuscript.

Efficiency of acry-DNA crosslinking.

Table S2.

Name	Sequence
Acry-DNA	ttttttggctgtaatcgg
Reported aptamer	CGACCGGTCTGGGGACCCTGTCTGGGTGTGTGGGTAGTAGGTCGtttt ttccgattacagcc
R5MPT	CGGGCGACGGGGATTTTTGGTGCACTATGTGGTTCGTTCGGGGTG GGctgcgttctccattctggttttttccgattacagcc
CSS.2	GGGACGACTAGCGTATGCGCCAGAAGTATACGAGGATAgtcgtcccttttt ccgattacagcc
TESS.1_trimmed	ACGGGATGTCCGGGGTACGGTGGTTGCAGTTCgttttttccgattacagcc
*Lowercase letters indicate the region corresponding to acry-DNA for immobilization, connected with the binding region of aptamer with polyT.	

Sequences in this work

Table S3

Aptamer	Method	T / ^oC	K_D / µM	Source
Ref	ITC	18	1.75<br>(ΔH=-29.9 kcal·mol^{-1}; ΔG=-7.67 kcal·mol^{-1})	B. Wang et al., 2022
		25	3.75<br>(ΔH=-34.5 kcal·mol^{-1}; ΔG=-7.40 kcal·mol^{-1})	
		30	3.13<br>(ΔH=-35.0 kcal·mol^{-1}; ΔG=-7.54 kcal·mol^{-1})	
		37	5.02<br>(ΔH=-39.3 kcal·mol^{-1}; ΔG=-7.52 kcal·mol^{-1})	
	OECT*	18	28.19	
		21	45.95	
		35	45.5	
		37	83.19	
R5MPT	ITC	18	5.47<br>(ΔH=-6.87 kcal·mol^{-1}; ΔG=-7.01 kcal·mol^{-1})	Unpublished work from our group
		25	10.2<br>(ΔH=-14.50 kcal·mol^{-1}; ΔG=-6.81 kcal·mol^{-1})	
		30	15<br>(ΔH=-10.20 kcal·mol^{-1}; ΔG=-6.69 kcal·mol^{-1})	
		37	15.1<br>(ΔH=-8.23 kcal·mol^{-1}; ΔG=-6.84 kcal·mol^{-1})	
	OECT	25	13.34	
		35	14.41	
	OECT^K	25	33	
CSS.2	FRET$^{\#}$	25	0.14	K.-A. Yang et al., 2017
	OECT	25	No OECT response	
TESS.1	FRET$^{\#}$	25	0.08	
	OECT	25	0.303	

* No OECT response for this aptamer at 25 – 30 ^oC.

$^{\#}$ Data from the source.

^{K} Kinetic measurement.

Affinities and thermodynamic parameters of the aptamers used in this work
